# Supplementary material for: Bio-Assay-Guided Isolation of Fractions and Constituents with Antioxidant and Lipid-lowering Activity from Allium cepa
Source: Antioxidants (Basel). 2023 Jul 18;12(7):1448. doi: 10.3390/antiox12071448 (PMC10376131; doi:10.3390/antiox12071448)
Supplement: Supplementary file 1 [file antioxidants-12-01448-s001.zip › antioxidants-2484827-supplementary.pdf]

# Figures S1-S65

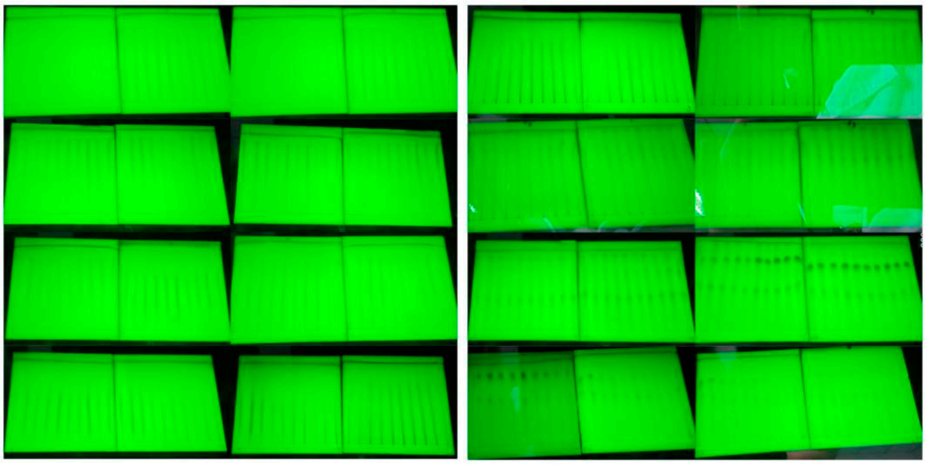

**Figure S1.** The pictures of the samples of AC30% after sephadex LH-20 column chromatography under 254 nm

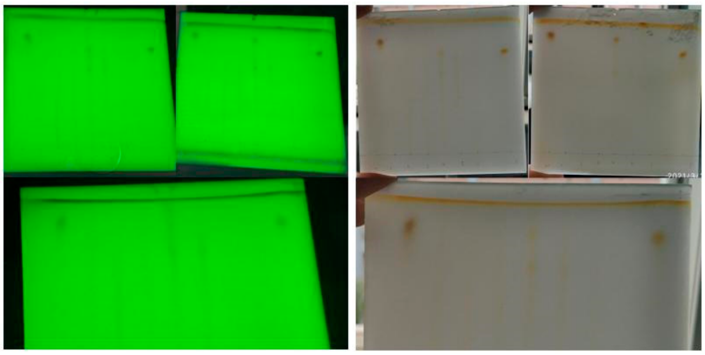

**Figure S2.** The TLC pictures of compounds Comp.1-Comp.5 (UV/iodine chromogen)

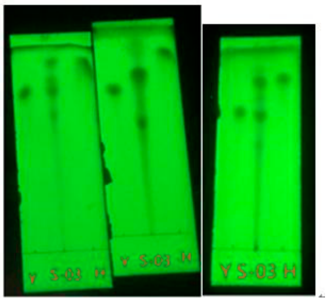


**Figure S3.** The TLC pictures of isoquercitrin (Y), sample Comp.3(S-03) and quercetin (H)

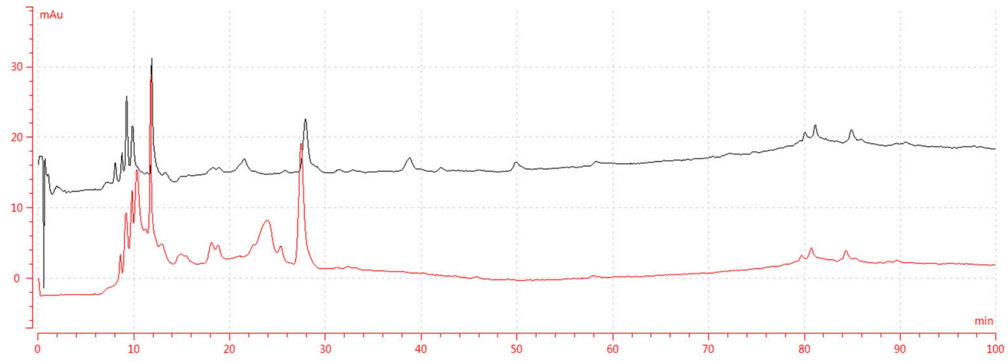


**Figure S4.** Total onion extract VS water fraction (black is the total onion extract chromatography under 254 nm)

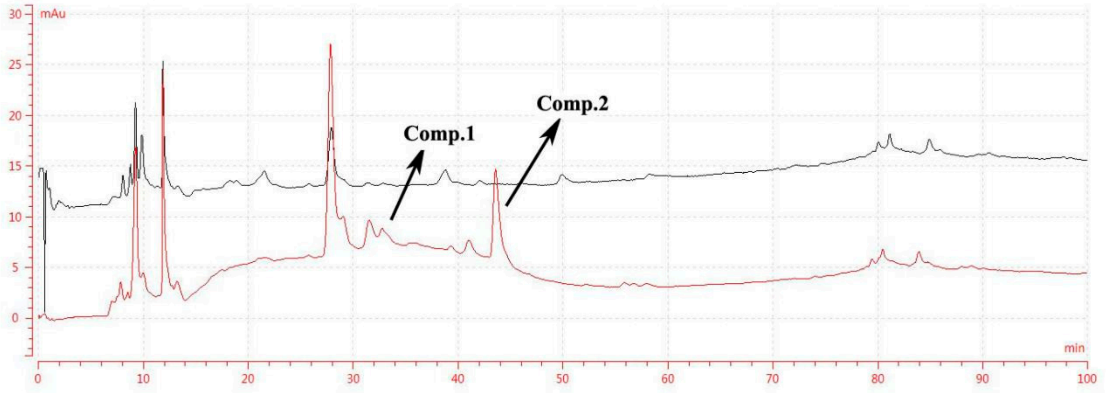


**Figure S5.** Total onion extract VS 30% ethanol fraction (black is the total onion extract chromatography under 254 nm)

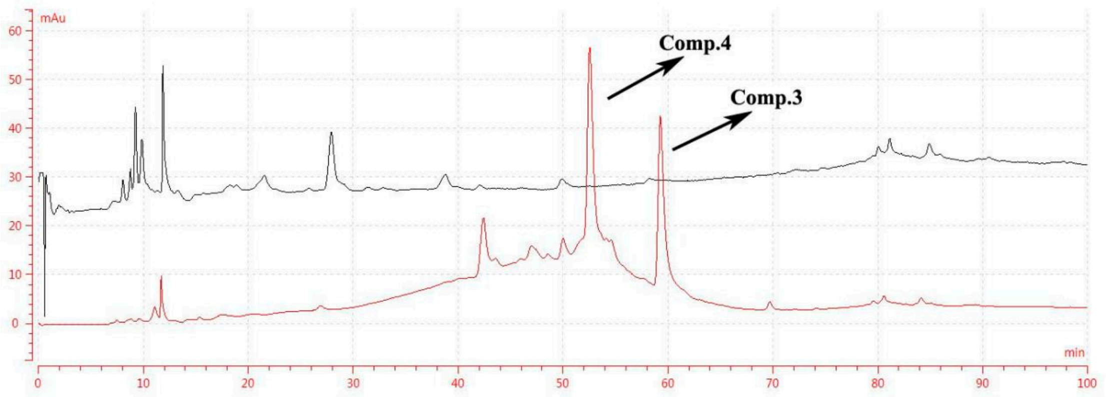


**Figure S6.** Total onion extract VS 50% ethanol fraction (black is the total onion extract chromatography under 254 nm)

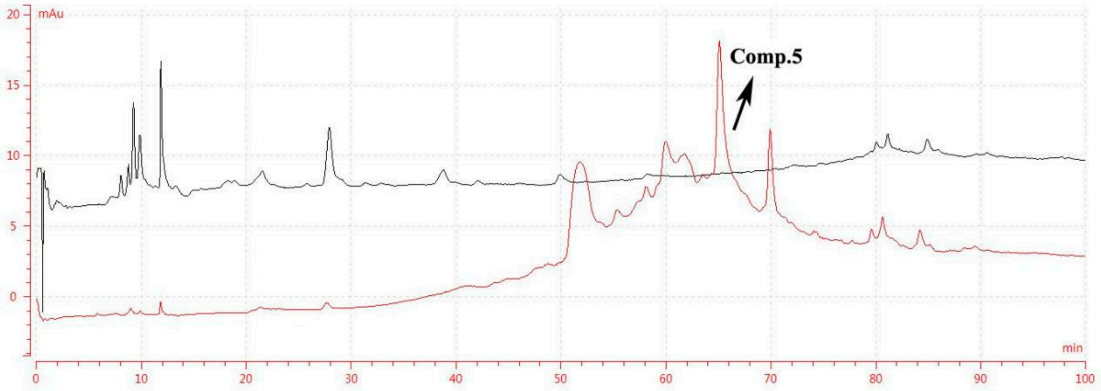


**Figure S7.** Total onion extract VS 70% ethanol fraction (black is the total onion extract chromatography under 254 nm)

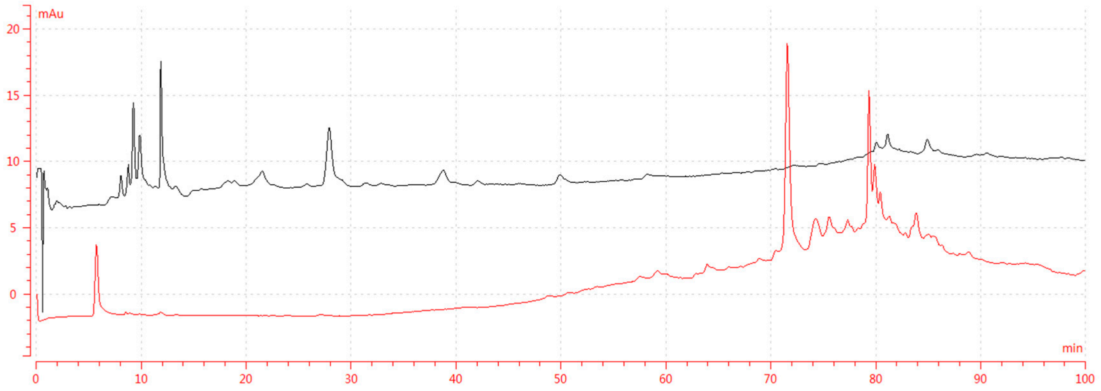


**Figure S8.** Total onion extract VS 95% ethanol fraction (black is the total onion extract chromatography under 254 nm)

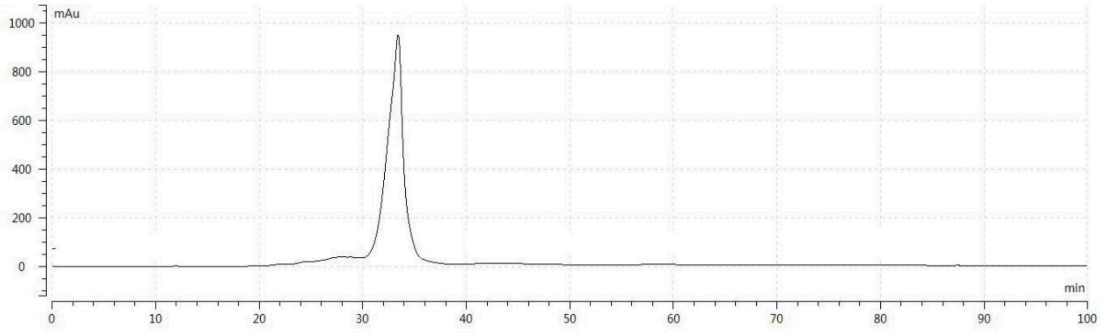


**Figure S9.** Liquid phase picture of compound 1

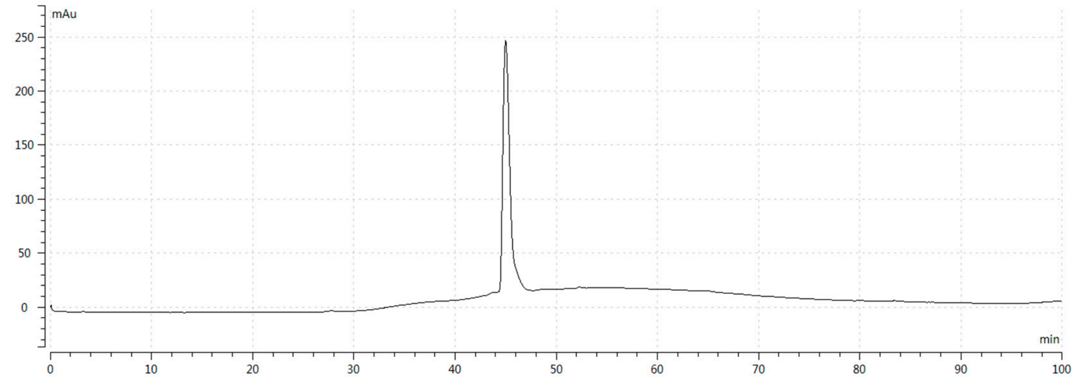


**Figure S10.** Liquid phase picture of compound 2

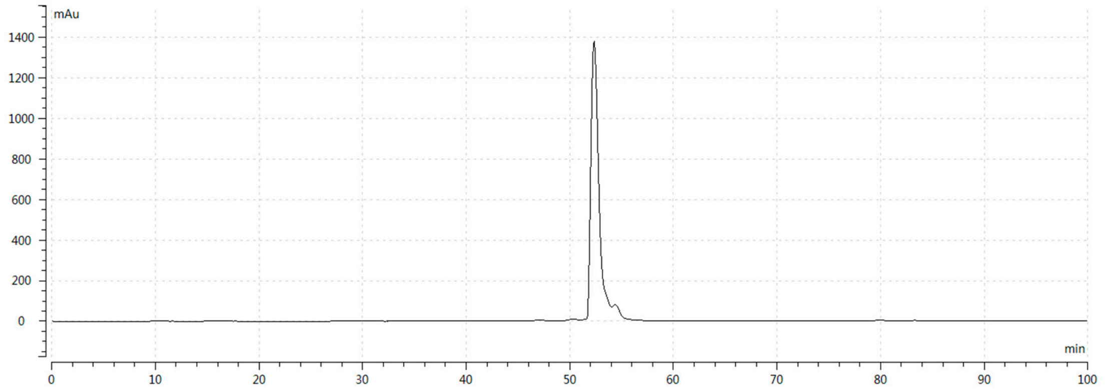


**Figure S11.** Liquid phase picture of compound 3

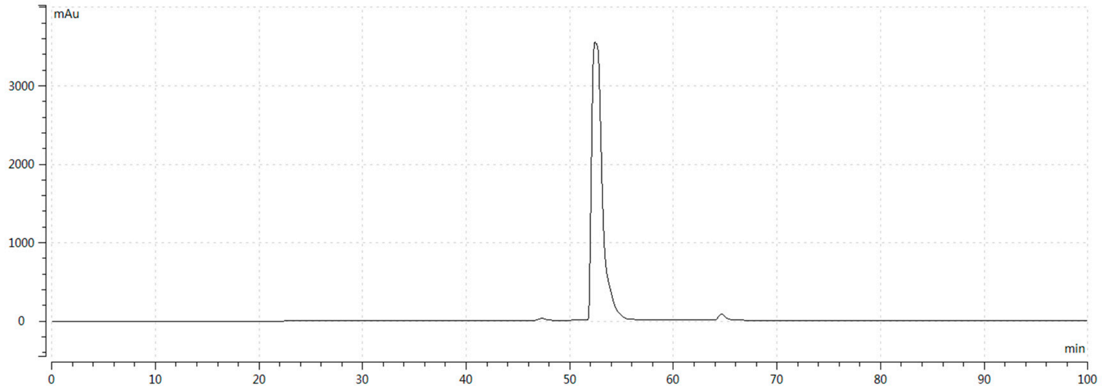


**Figure S12.** Liquid phase picture of compound 3 mixed with isoquercitrin

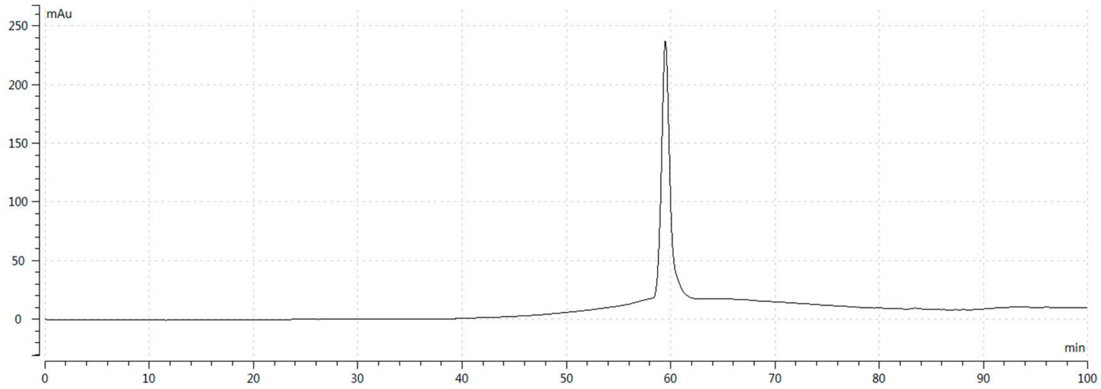


**Figure S13.** Liquid phase picture of compound 4

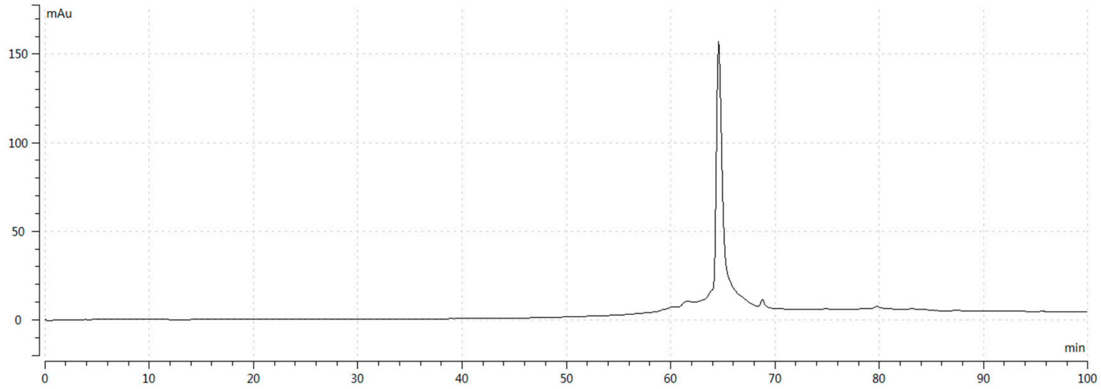


**Figure S14.** Liquid phase picture of compound 5

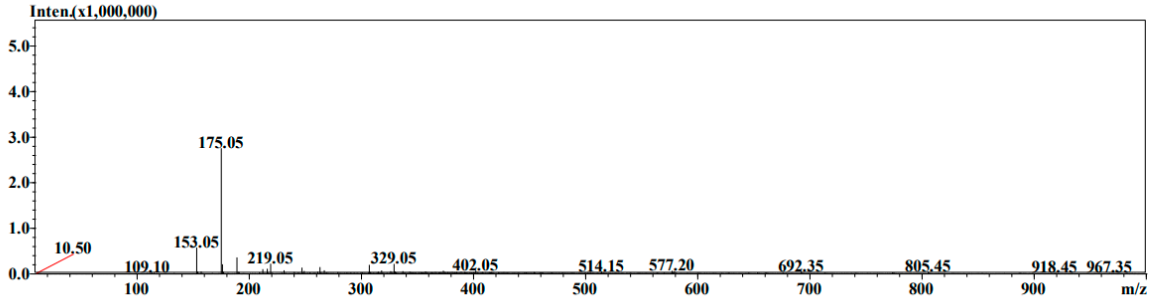


**Figure S15.** Mass spectrogram of compound 1

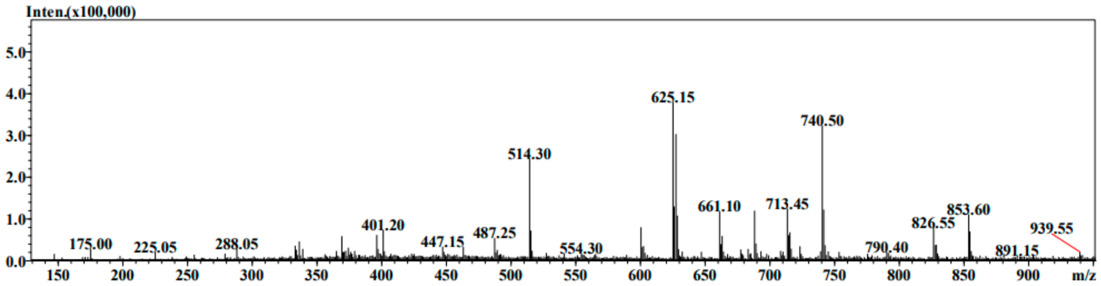


**Figure S16.** Mass spectrogram of compound 2

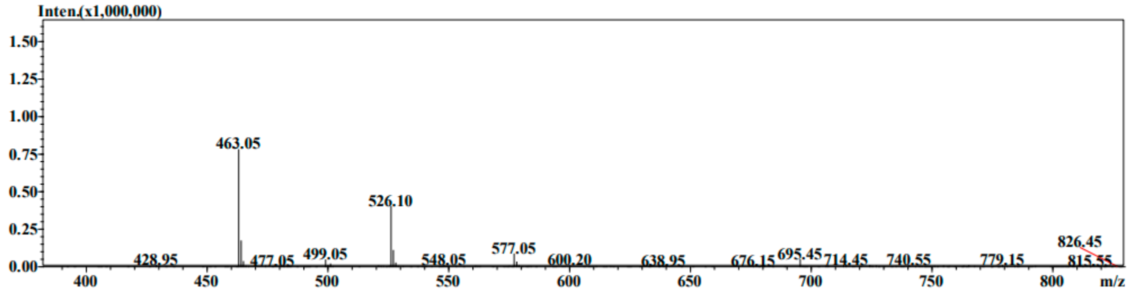


**Figure S17.** Mass spectrogram of compound 3

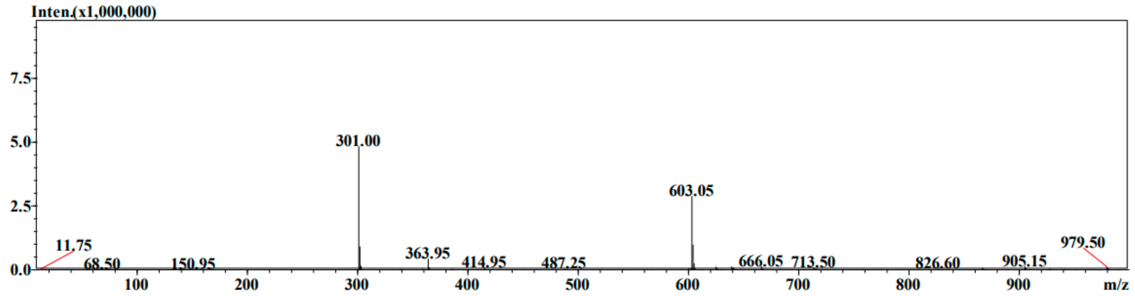


**Figure S18.** Mass spectrogram of compound 4

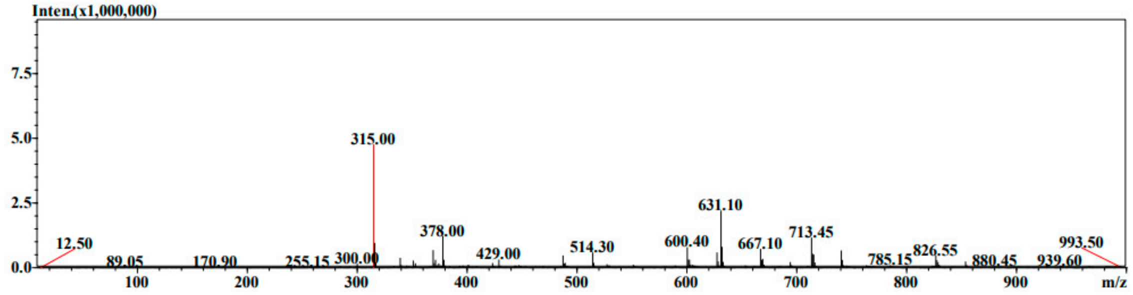


**Figure S19.** Mass spectrogram of compound 5

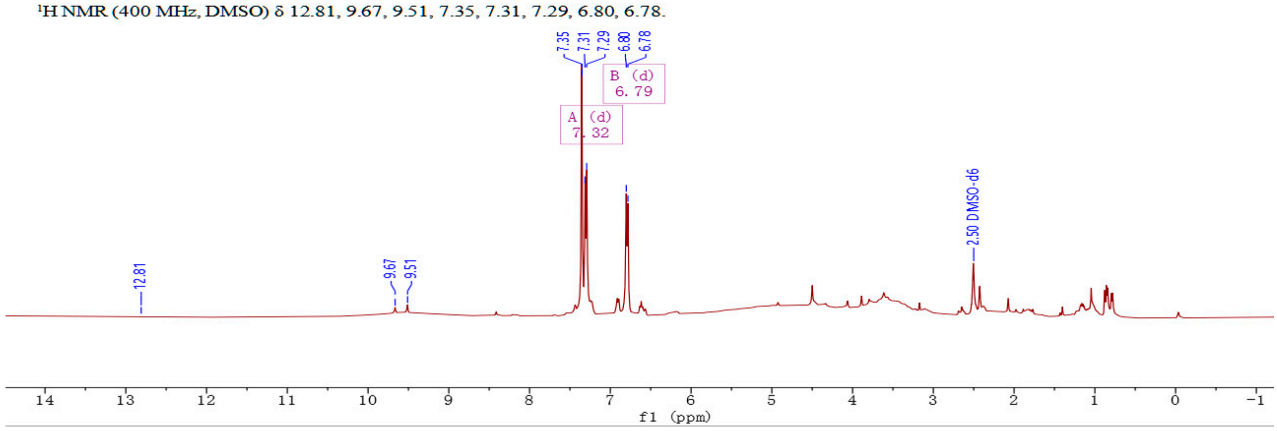


**Figure S20.** ¹H-NMRspectrum of compound 1

$^{13}$C NMR (101 MHz, DMSO) δ 167.70, 150.29, 145.13, 122.25, 121.93, 116.85, 115.43.

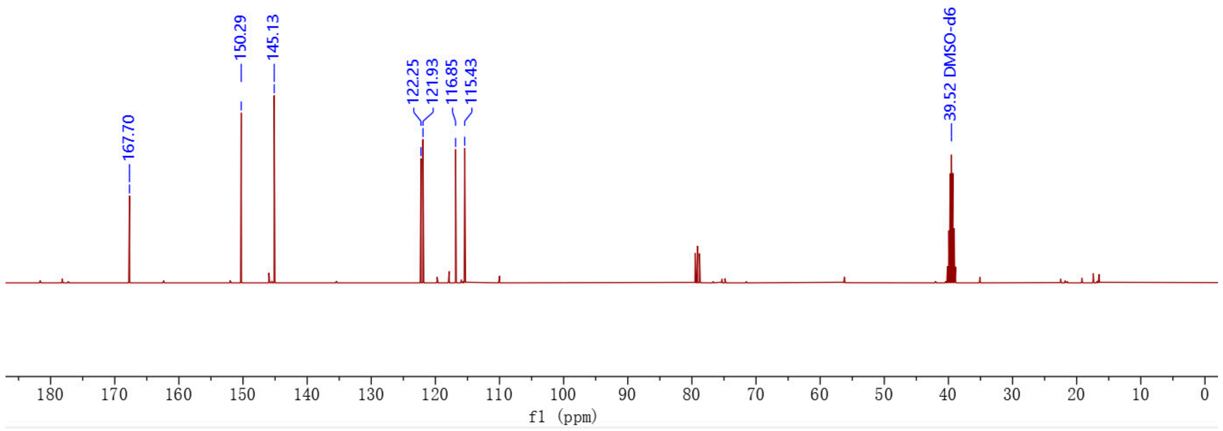


**Figure S21.** $^{13}$C-NMR spectrum of compound 1

$^{1}$H NMR (400 MHz, DMSO) δ 1.19 (s, 1H), 1.27 – 1.33 (m, 1H), 2.56 (d, $J$ = 2.3 Hz, 7H), 3.15 (d, $J$ = 4.3 Hz, 2H), 3.27 (d, $J$ = 6.8 Hz, 4H), 3.37 – 3.49 (m, 16H), 3.56 (s, 2H), 3.62 – 3.68 (m, 2H), 3.79 (d, $J$ = 11.7 Hz, 1H), 4.37 (s, 1H), 4.69 (s, 1H), 4.93 (d, $J$ = 6.9 Hz, 1H), 5.03 (s, 1H), 5.15 (s, 4H), 5.37 (s, 1H), 5.48 – 5.52 (m, 1H), 5.56 (d, $J$ = 6.9 Hz, 1H), 6.28 (s, 1H), 6.51 (s, 1H), 7.27 (d, $J$ = 8.6 Hz, 1H), 7.67 (d, $J$ = 8.6 Hz, 1H), 7.71 (s, 1H), 8.97 (s, 1H), 11.00 (s, 1H), 12.64 (s, 1H).

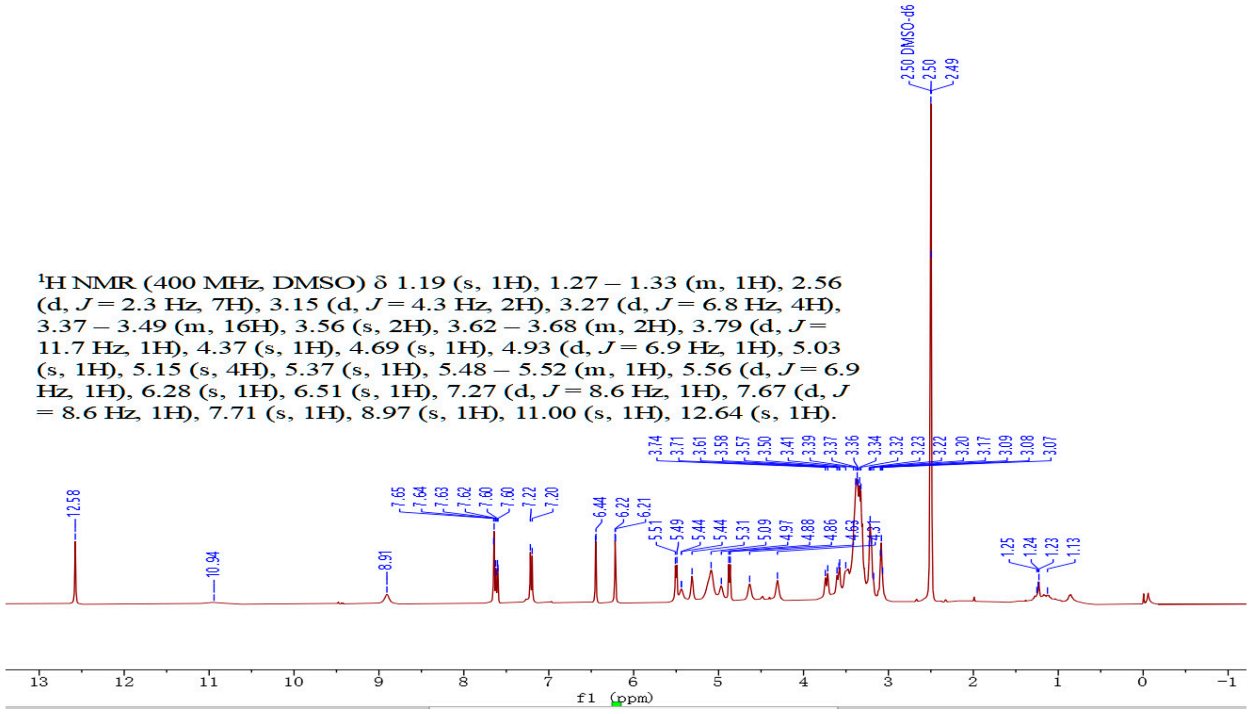


**Figure S22.** $^{1}$H-NMR spectrum of compound 2

13C NMR (101 MHz, DMSO) δ 177.64, 164.35, 161.32, 156.51, 155.58, 147.60, 146.19, 133.90, 124.51, 121.11, 116.61, 115.46, 104.20, 101.45, 100.78, 98.84, 93.78, 77.75, 77.30, 76.55, 75.92, 74.22, 73.36, 70.05, 69.80, 61.04, 60.76.

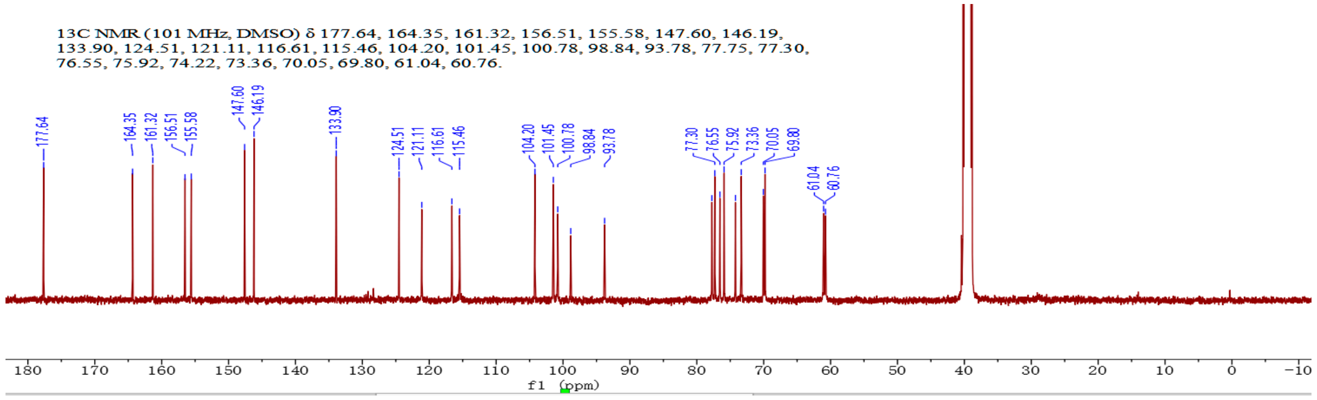


**Figure S23.** $^{13}$C-NMR spectrum of compound 2

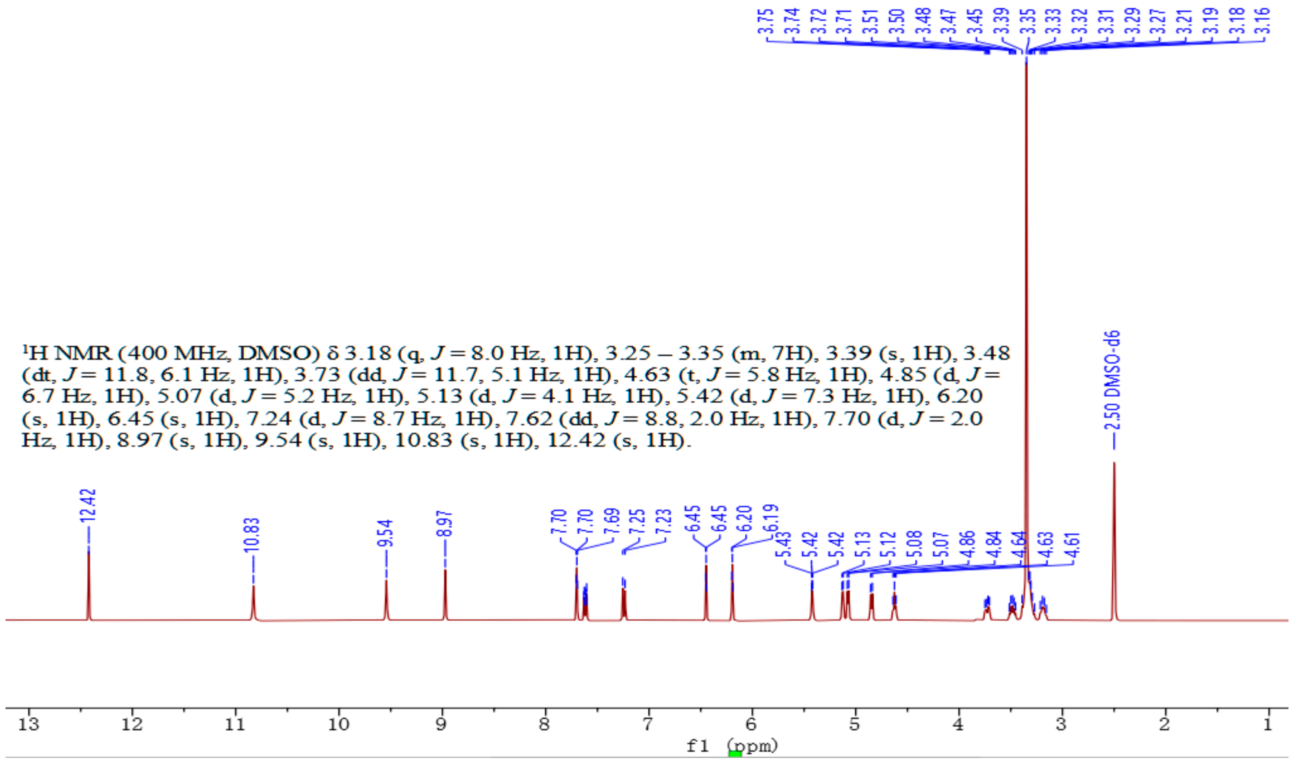


**Figure S24.** $^{1}$H-NMR spectrum of compound 3

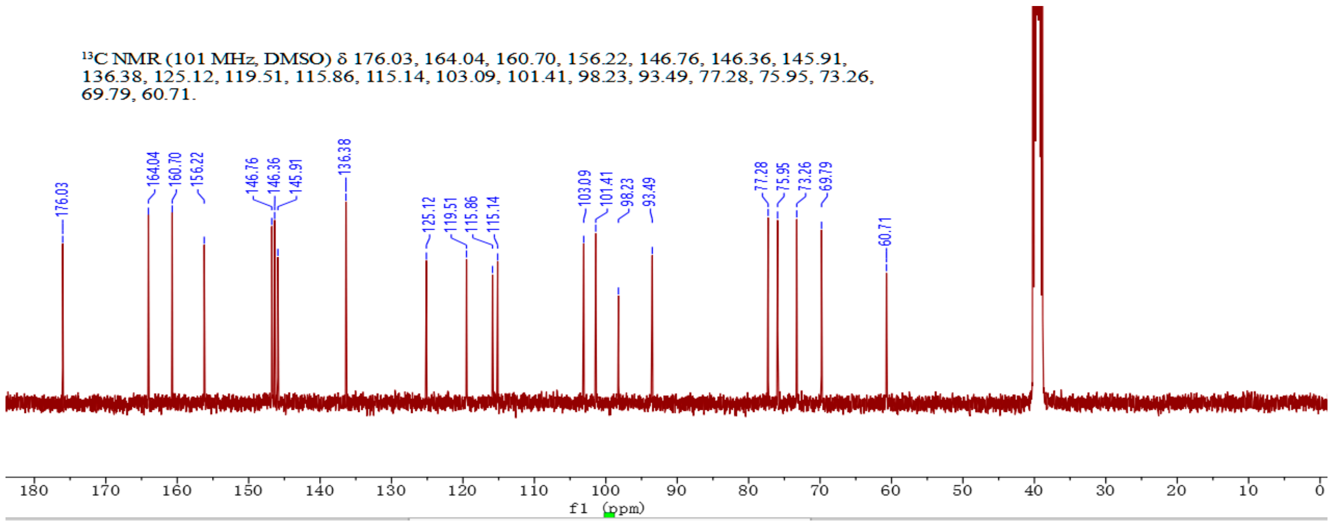


**Figure S25.** $^{13}$C-NMR spectrum of compound 3

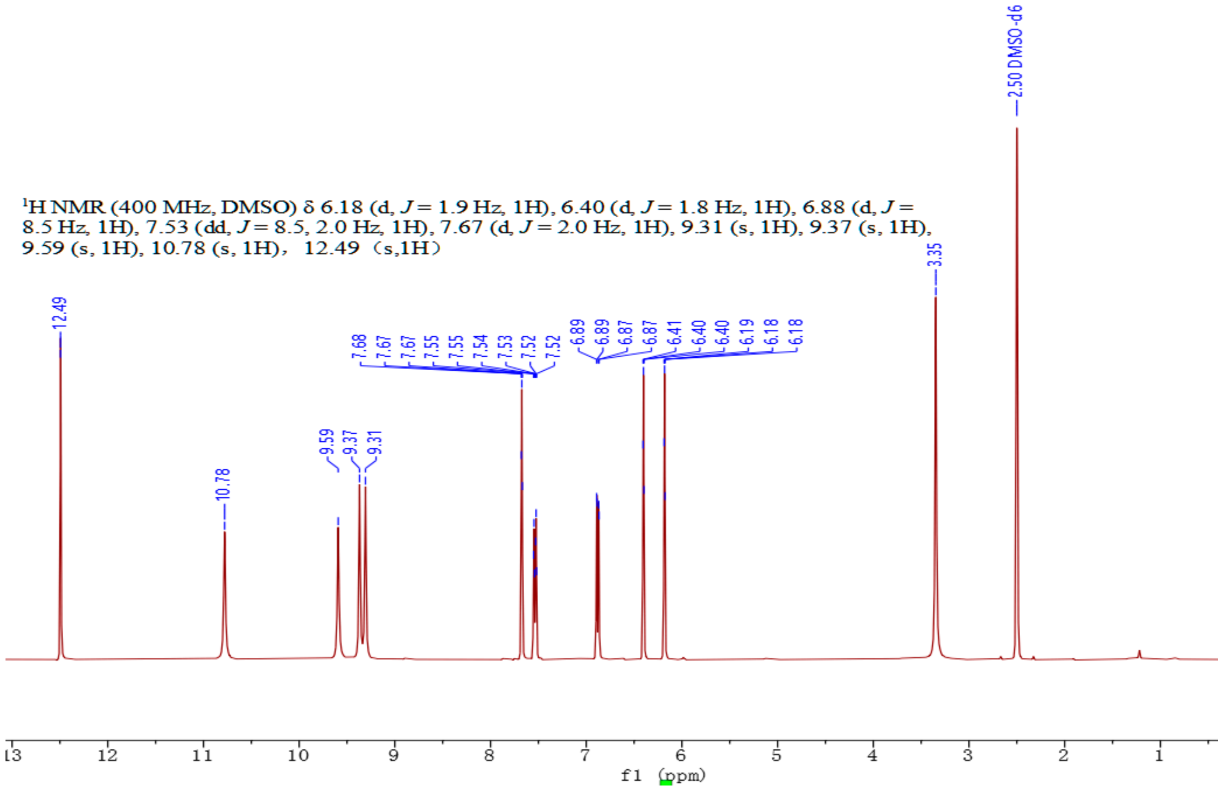


**Figure S26.** $^1$H-NMR spectrum of compound 4

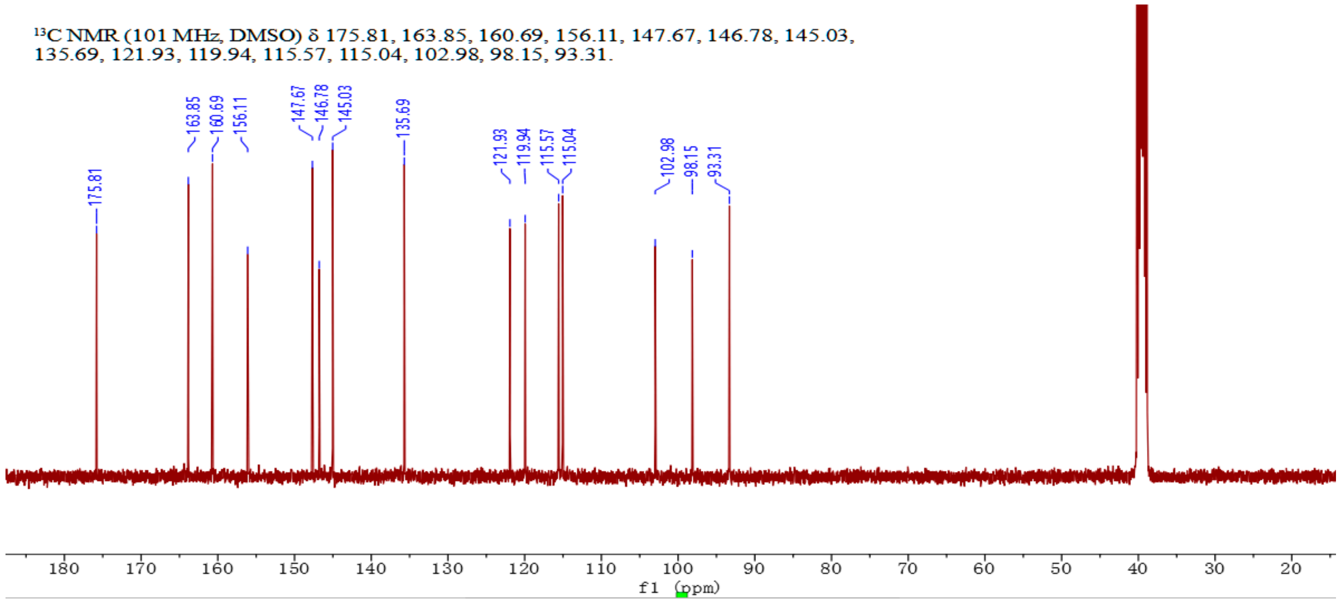


**Figure S27.** $^{13}$C-NMR spectrum of compound 4

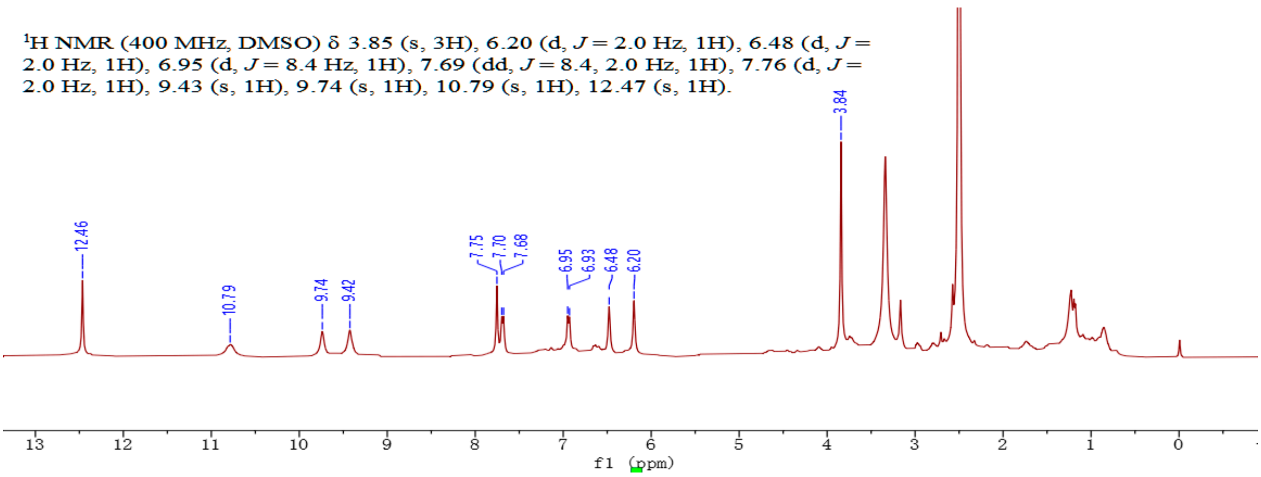


**Figure S28.** $^{1}$H-NMR spectrum of compound 5

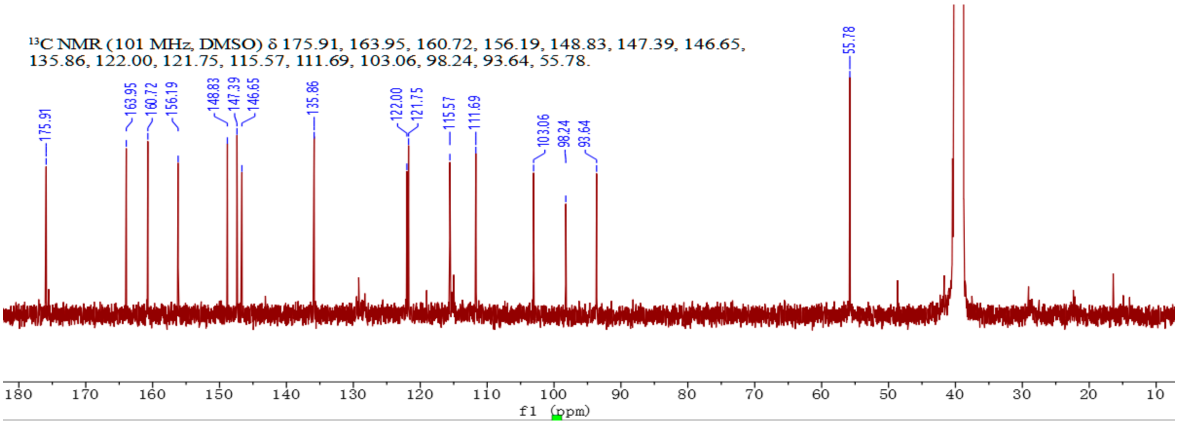


**Figure S29.** $^{13}$C-NMR spectrum of compound 5

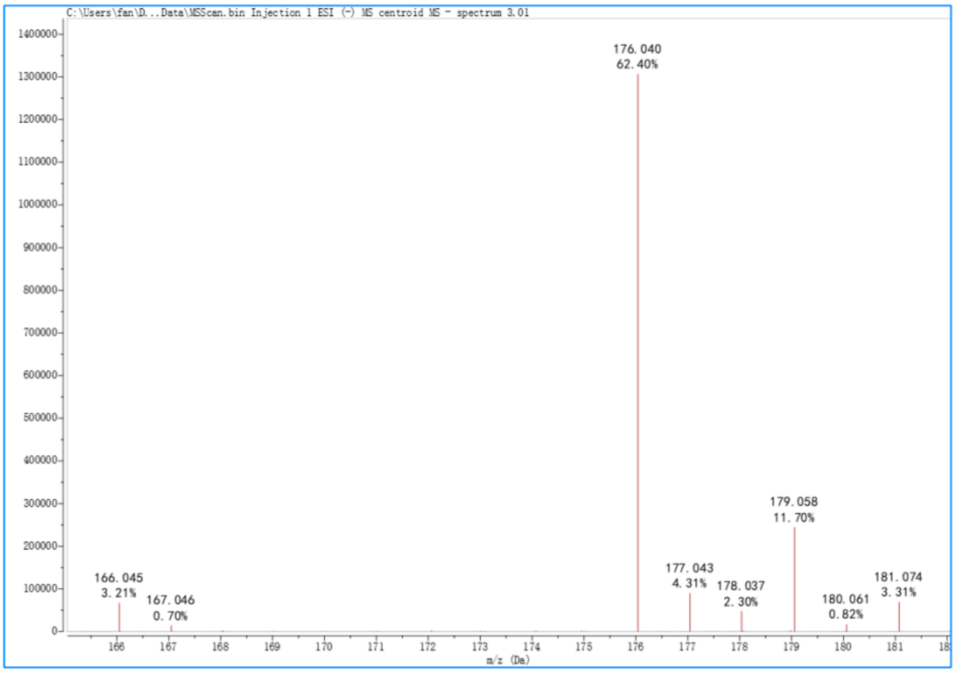


**Figure S30.** The first-order mass spectrum of compound A

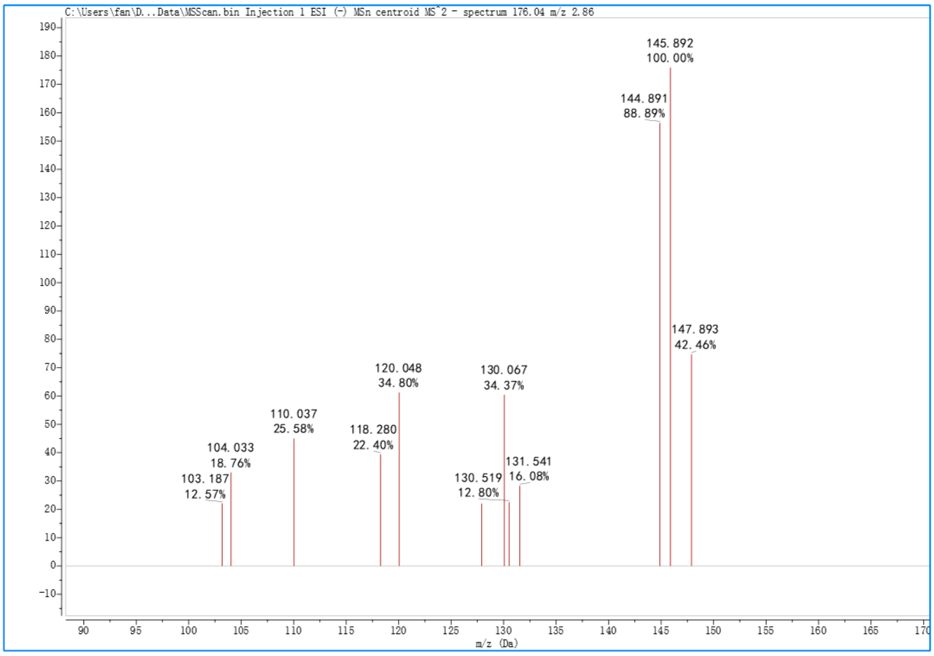


**Figure S31.** Secondary mass spectrum of compound A

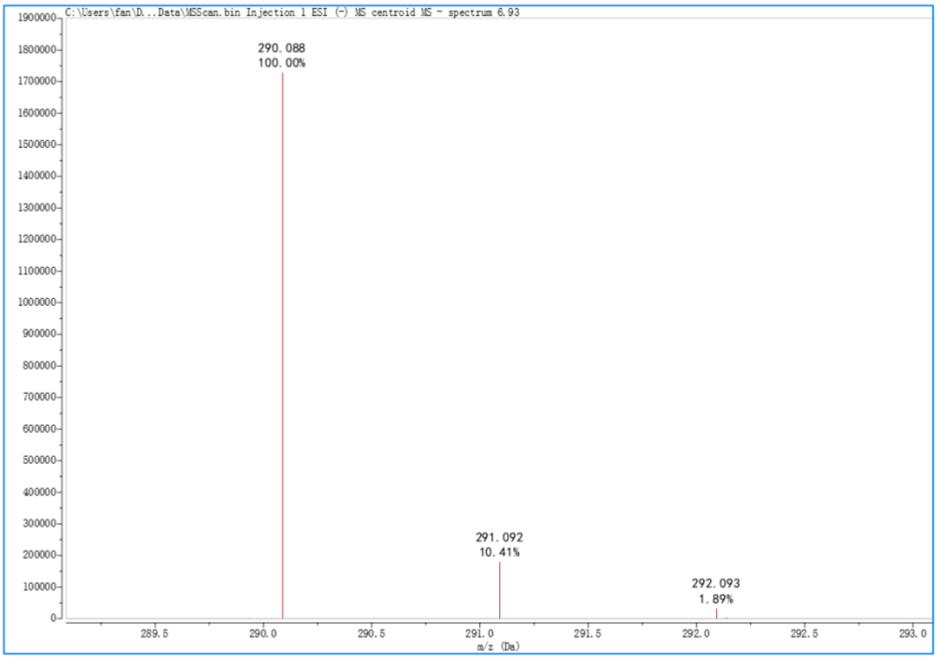


**Figure S32.** The first-order mass spectrum of compound B

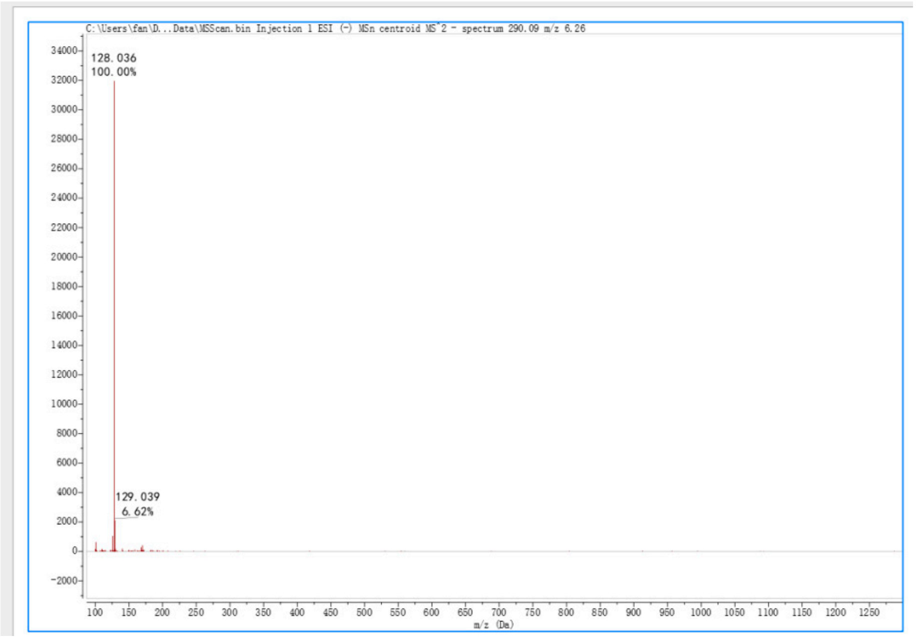


**Figure S33.** Secondary mass spectrum of compound B

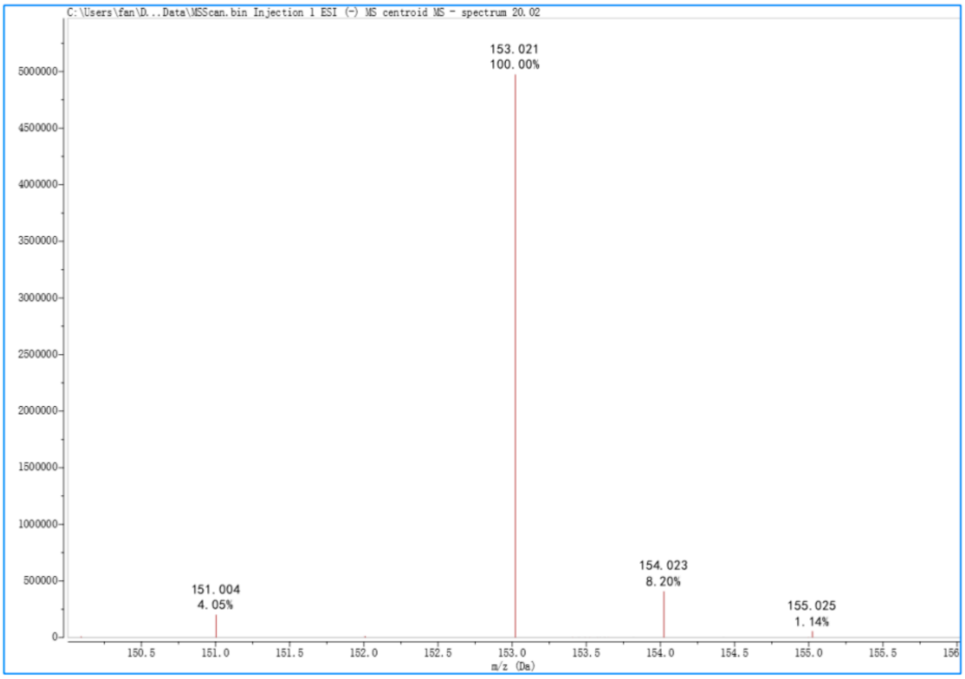


**Figure S34.** The first-order mass spectrum of compound C

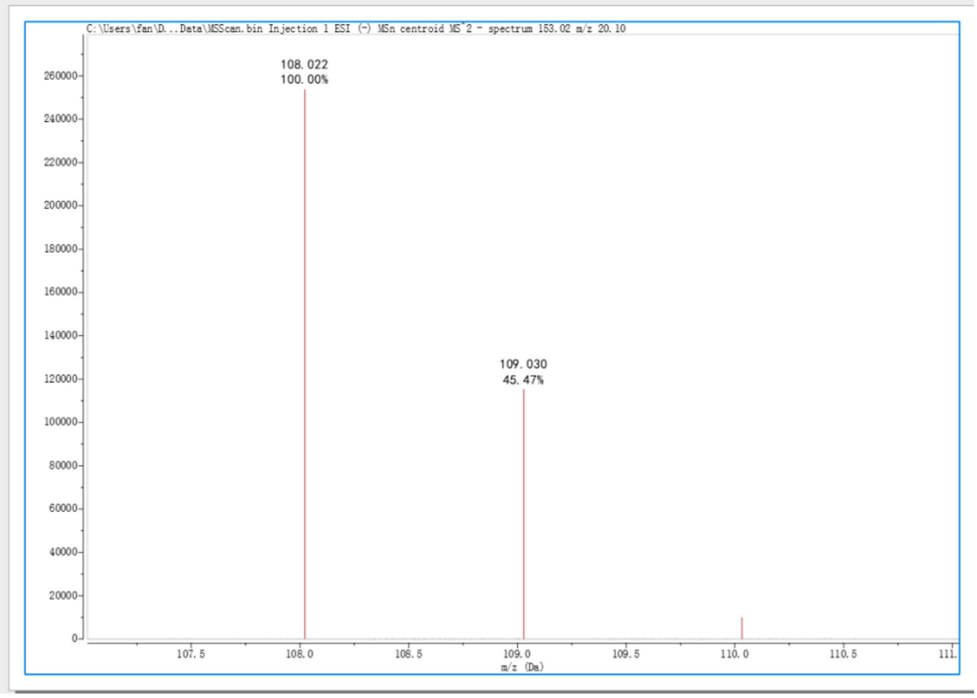


**Figure S35.** Secondary mass spectrum of compound C

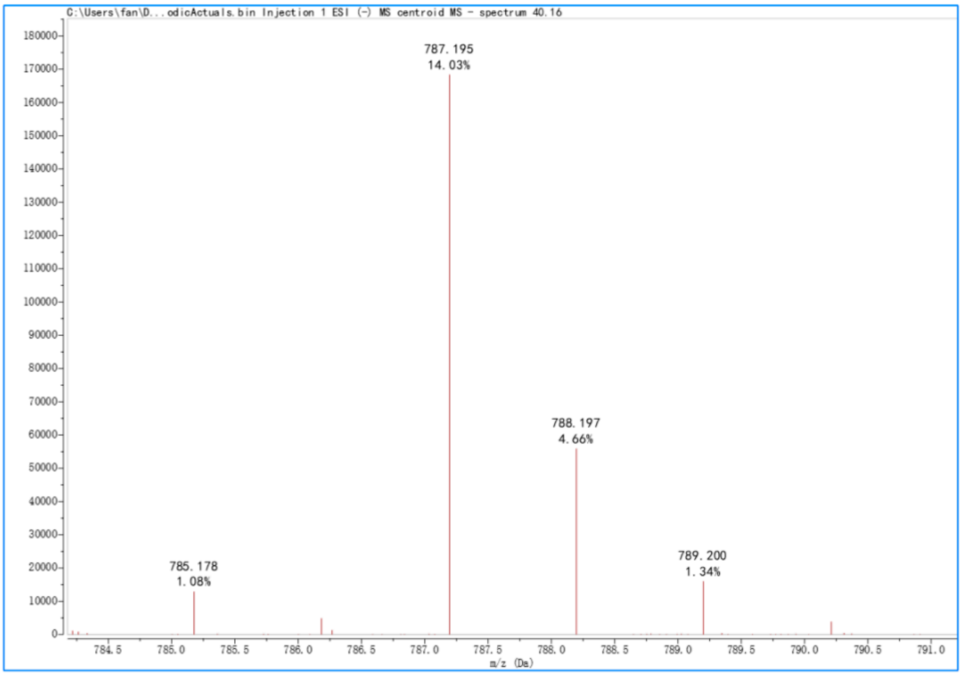


**Figure S36.** The first-order mass spectrum of compound D

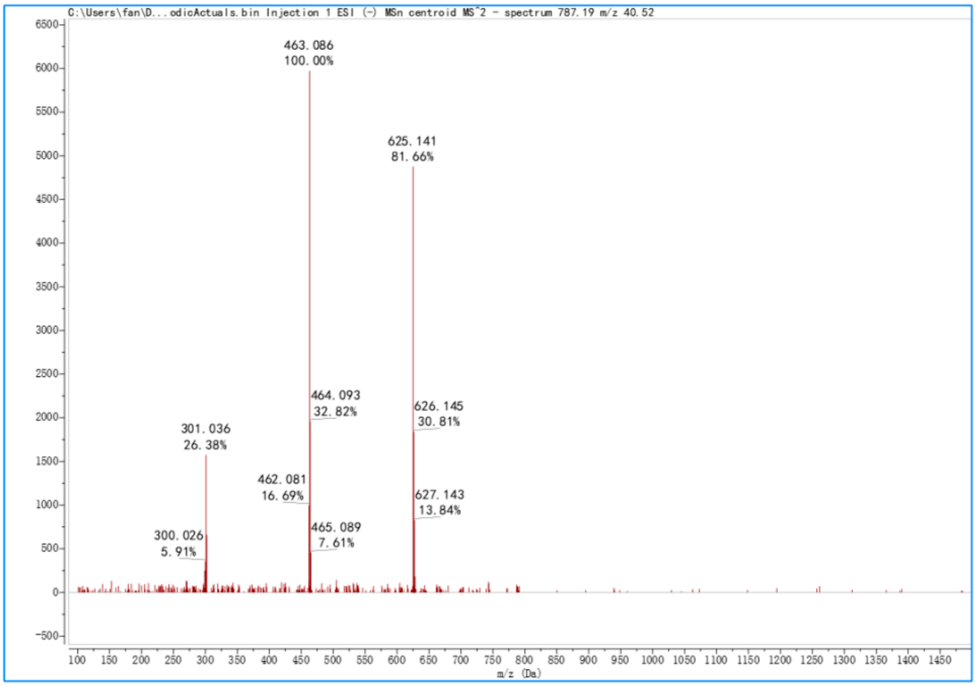


**Figure S37.** Secondary mass spectrum of compound D

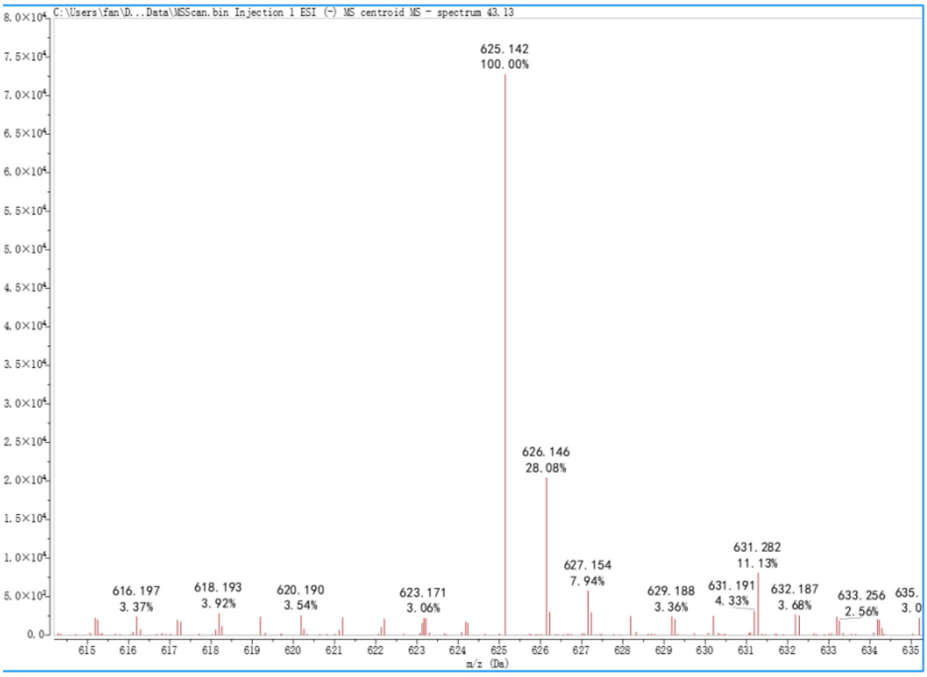


**Figure S38.** The first-order mass spectrum of compound E

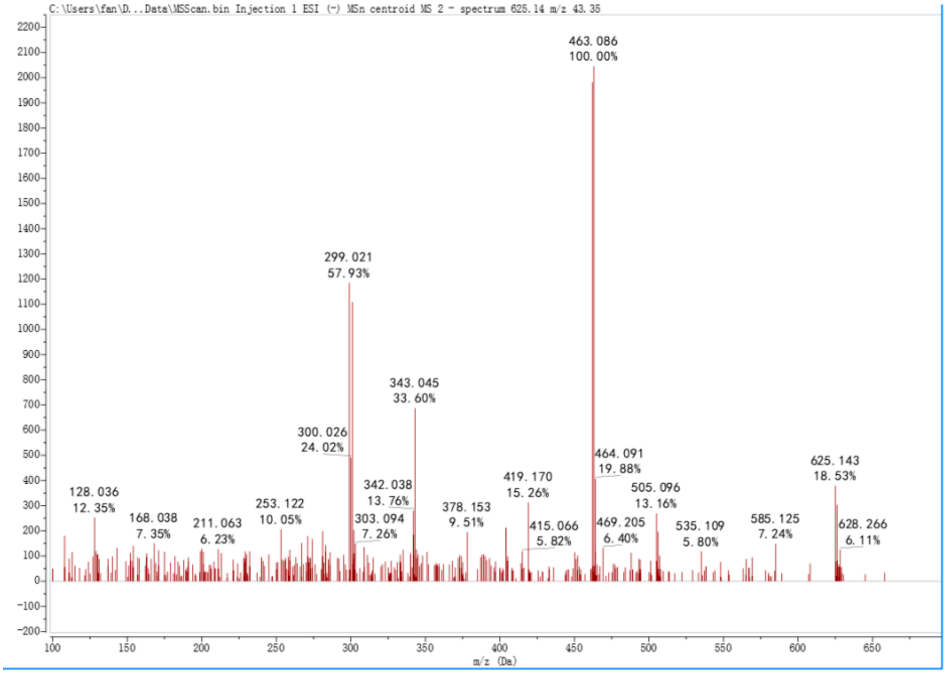


**Figure S39.** Secondary mass spectrum of compound E

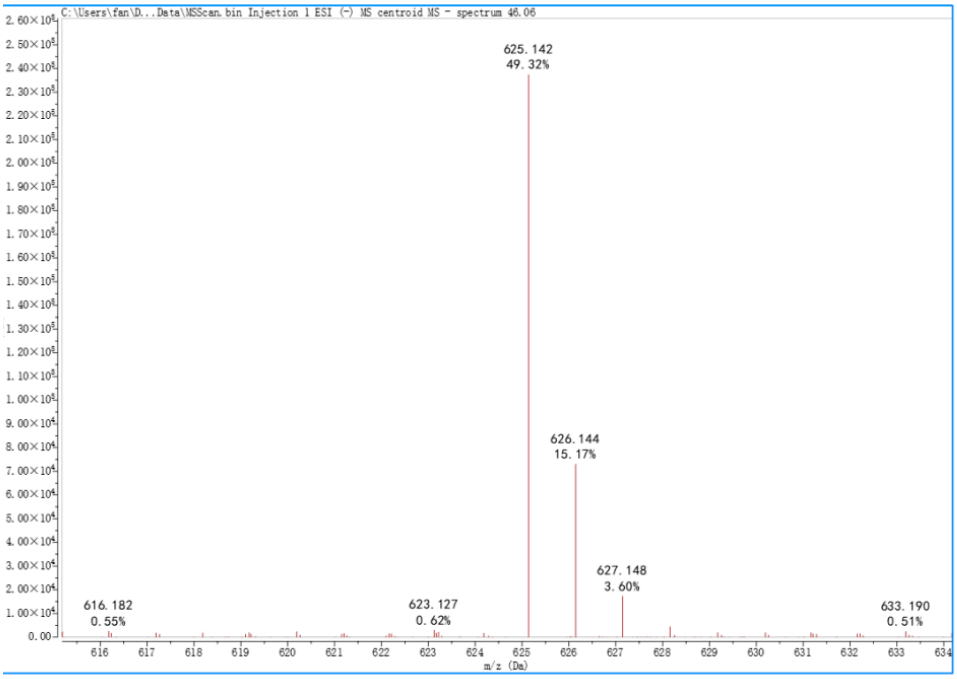


**Figure S40.** The first-order mass spectrum of compound F

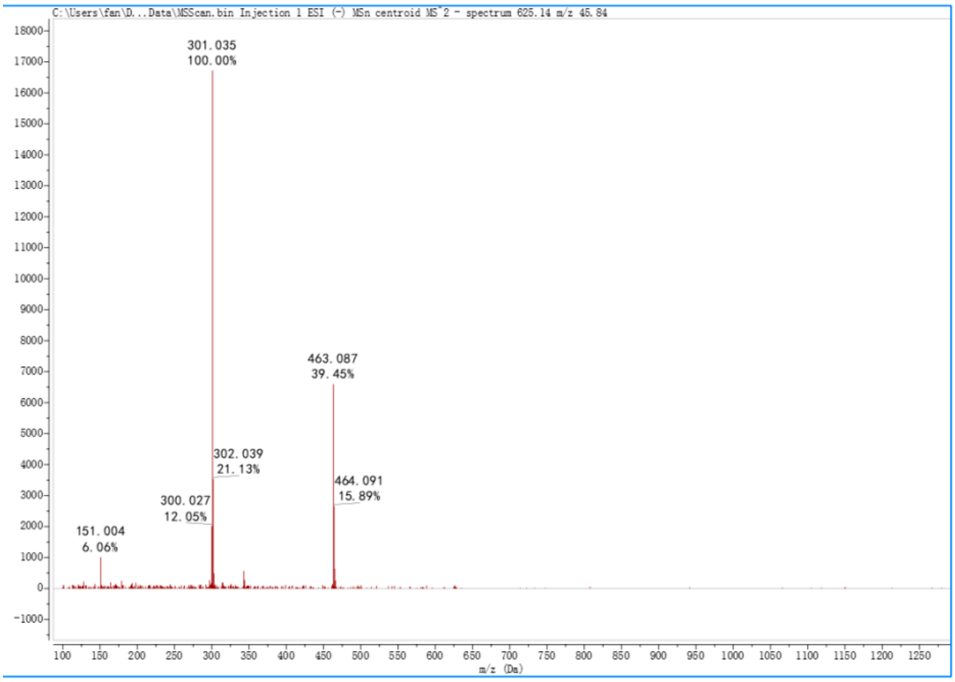


**Figure S41.** Secondary mass spectrum of compound F

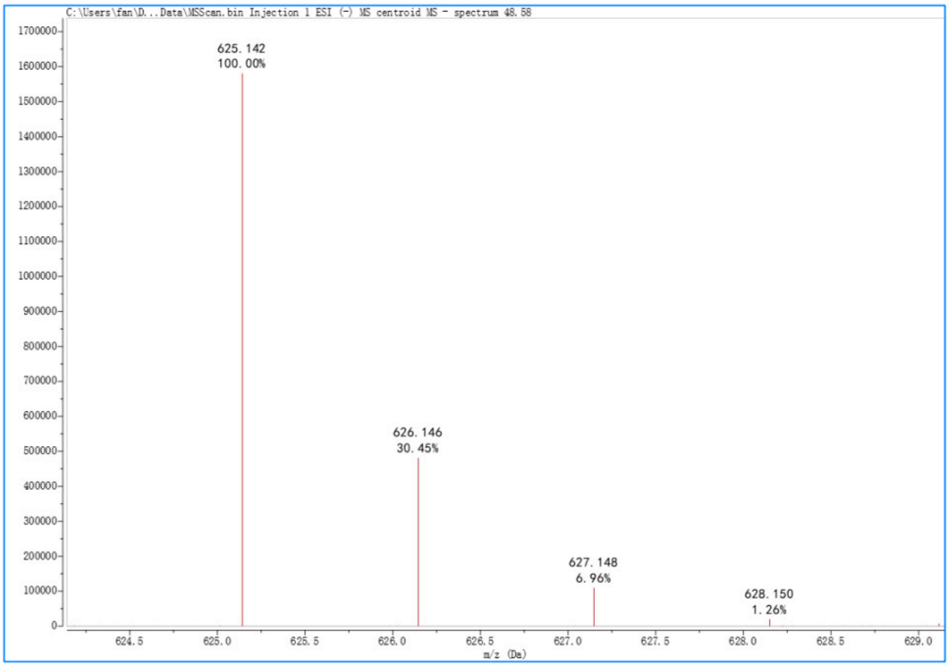


**Figure S42.** The first-order mass spectrum of compound G

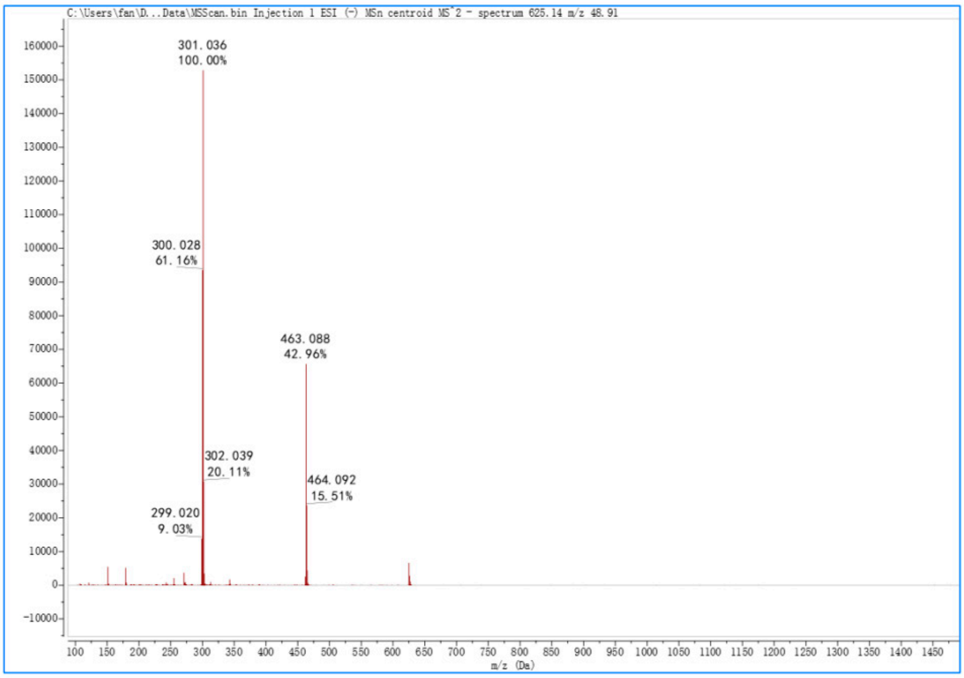


**Figure S43.** Secondary mass spectrum of compound G

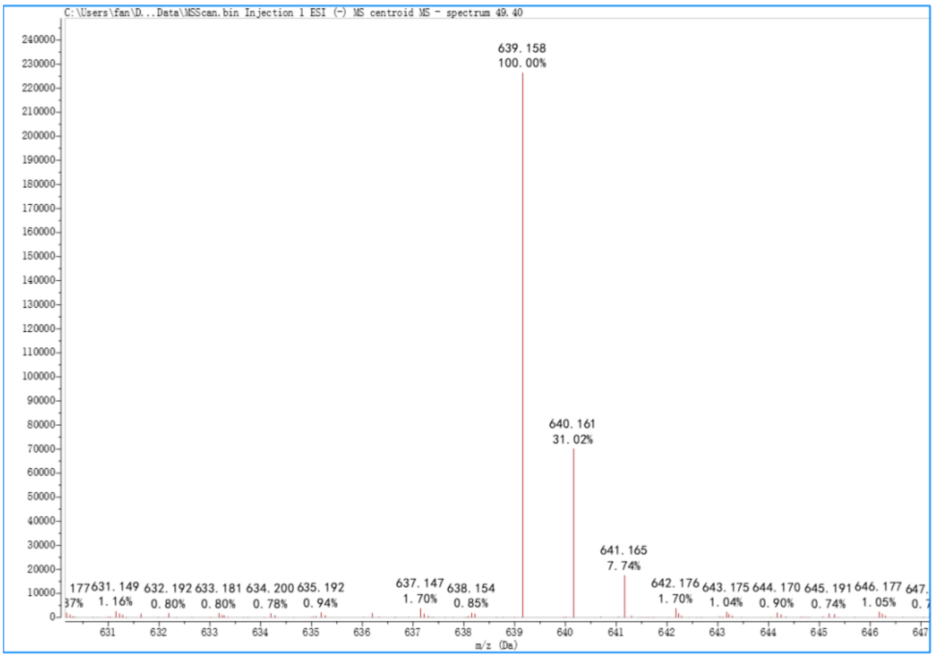


**Figure S44.** The first-order mass spectrum of compound H

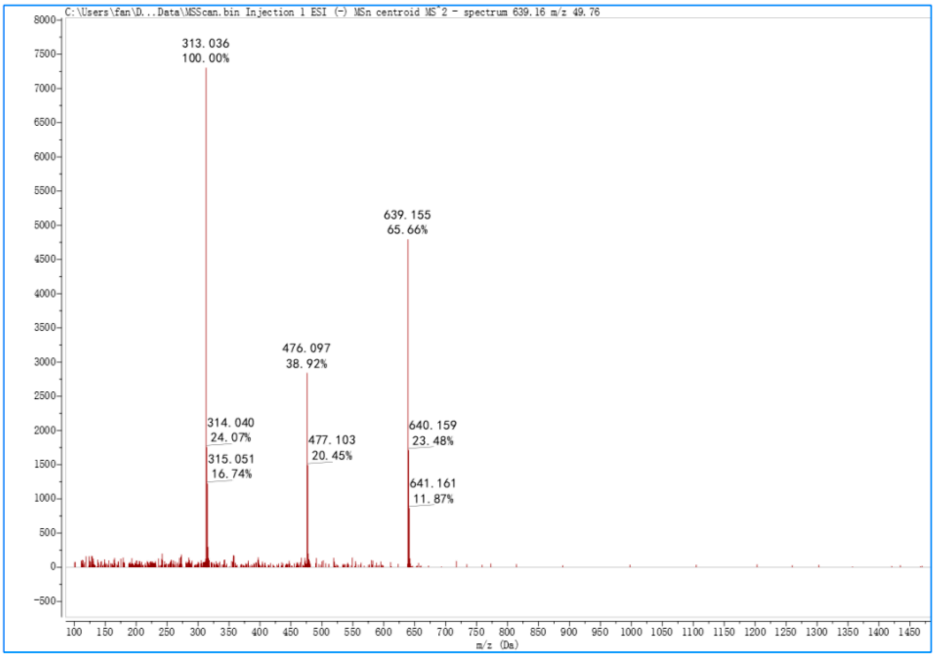


**Figure S45.** Secondary mass spectrum of compound H

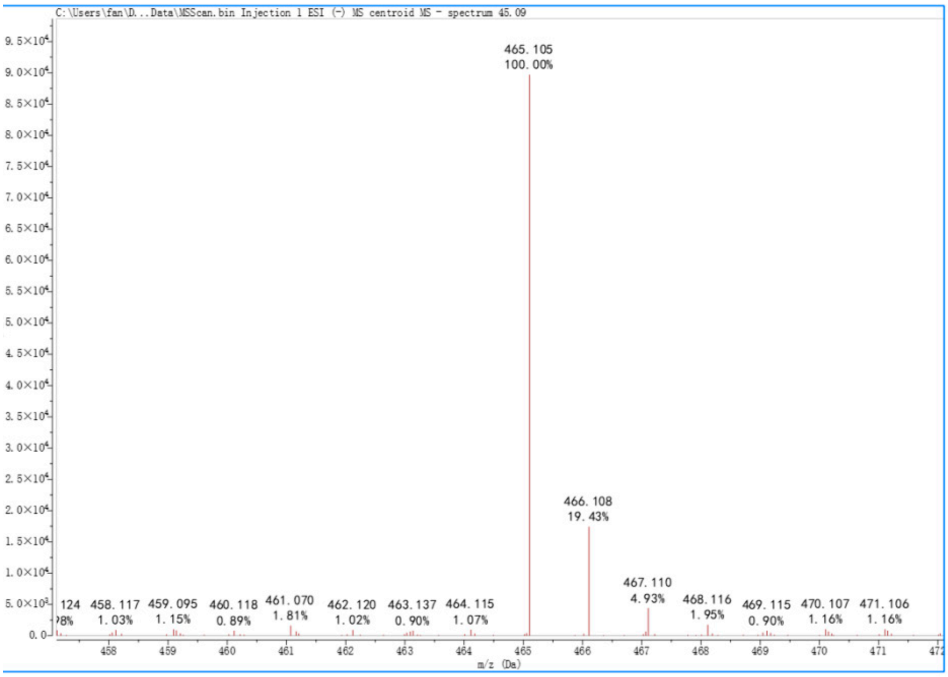


**Figure S46.** The first-order mass spectrum of compound Ⅰ

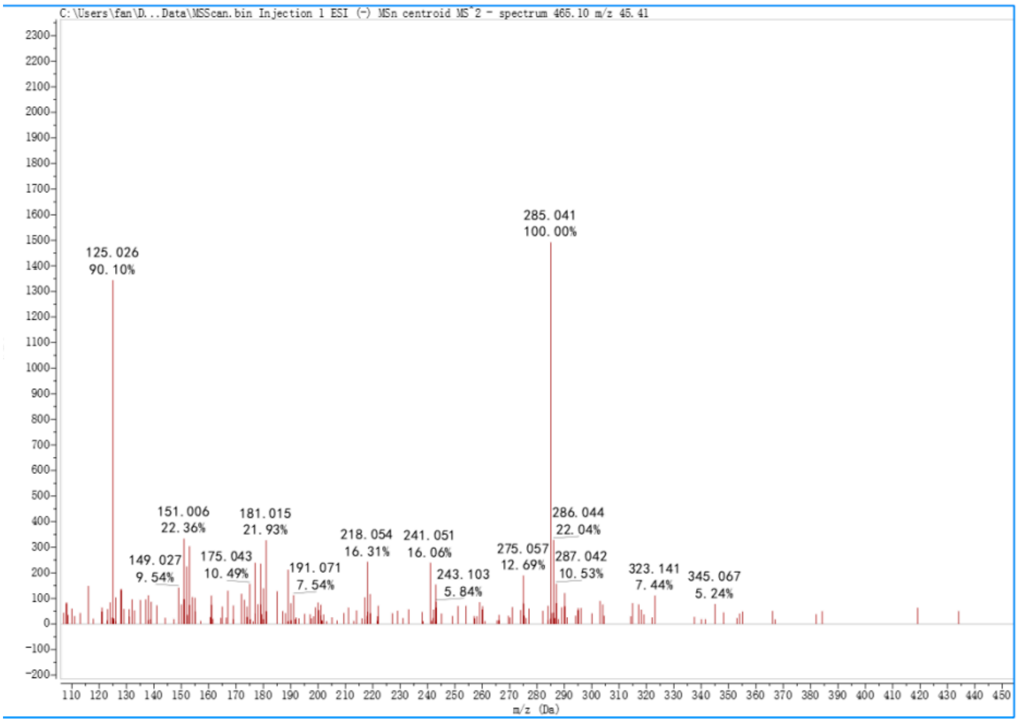


**Figure S47.** Secondary mass spectrum of compound Ⅰ

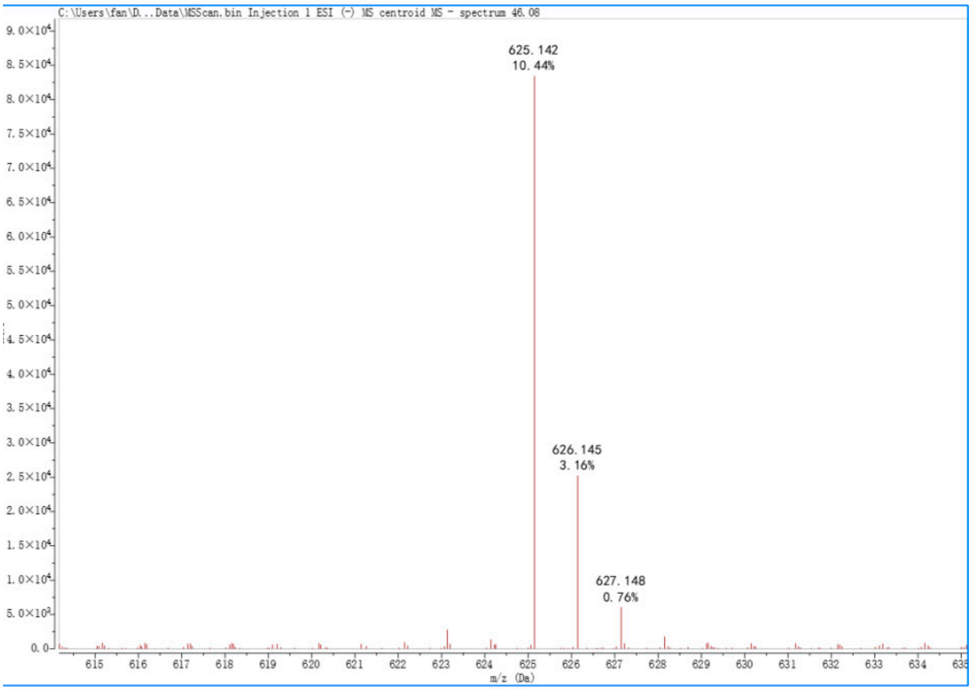


**Figure S48.** The first-order mass spectrum of compound Ⅱ

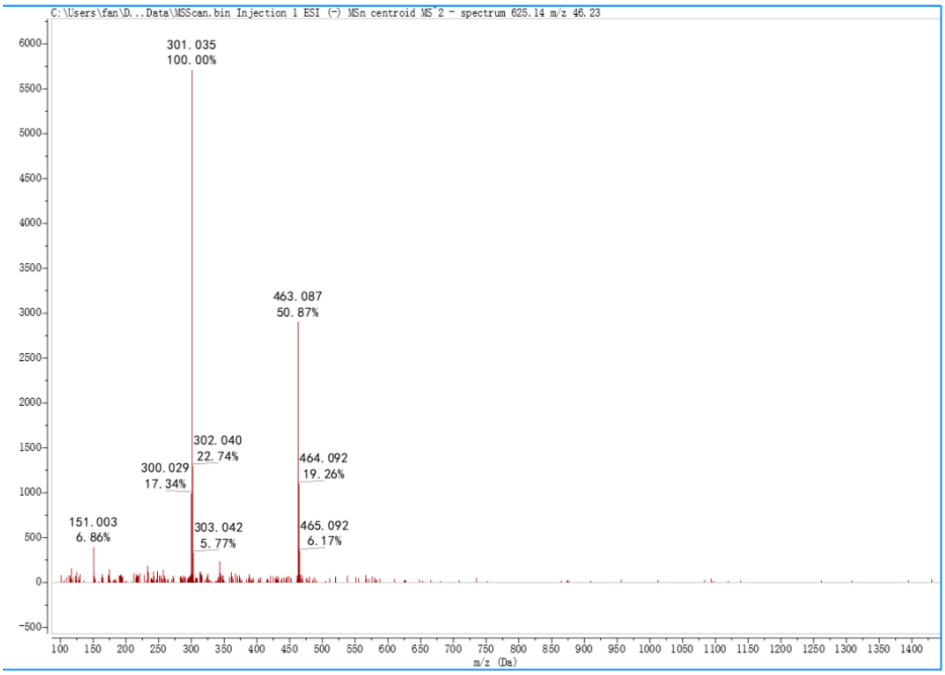


**Figure S49.** Secondary mass spectrum of compound Ⅱ

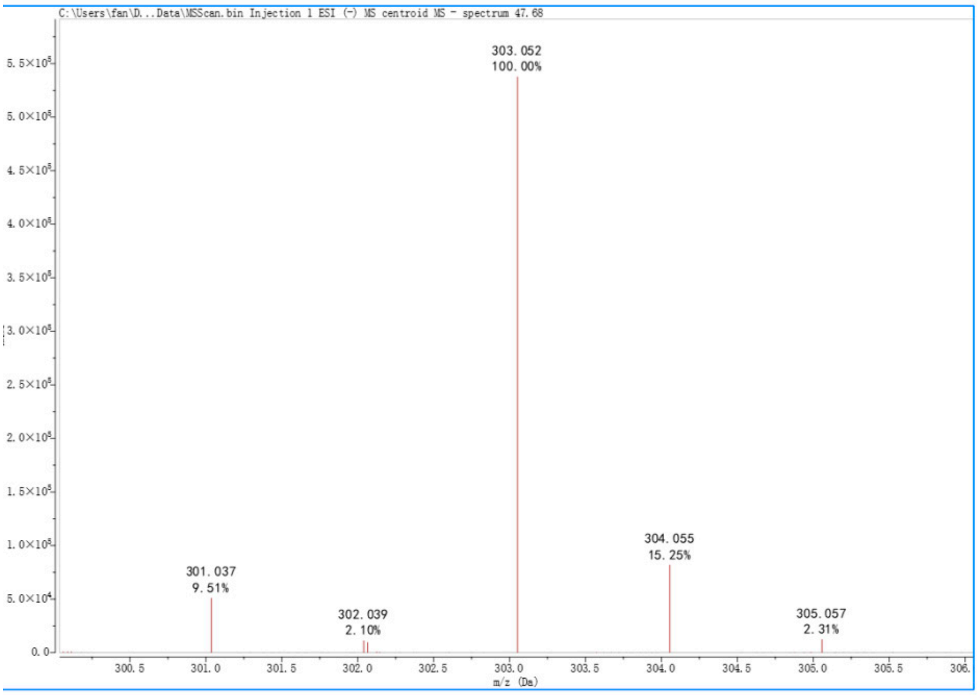


**Figure S50.** The first-order mass spectrum of compound Ⅲ

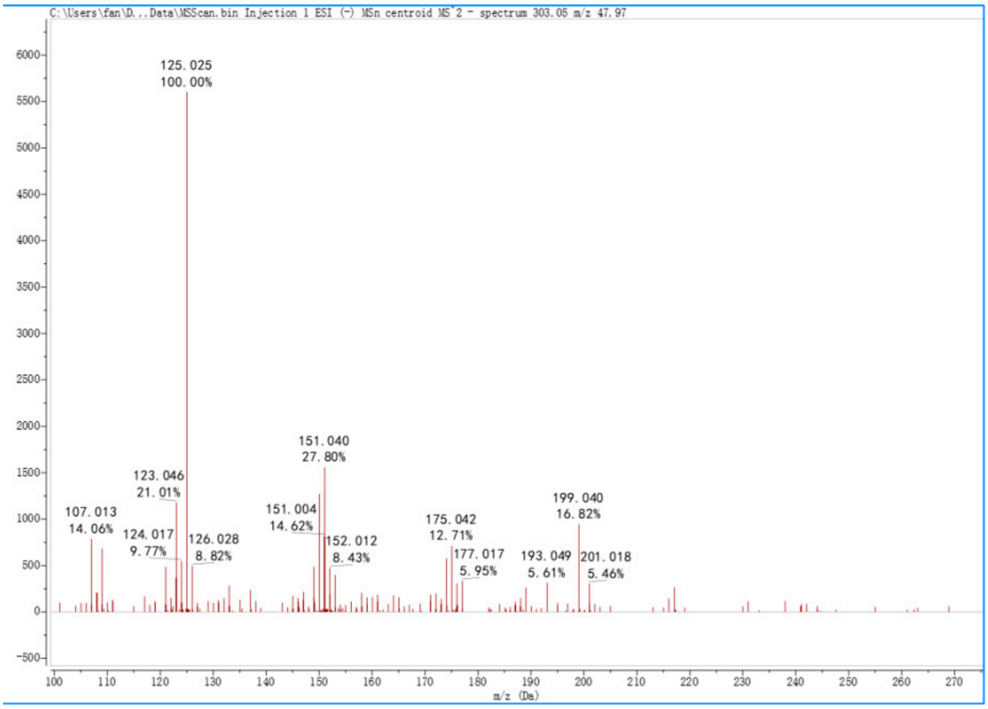


**Figure S51.** Secondary mass spectrum of compound Ⅲ

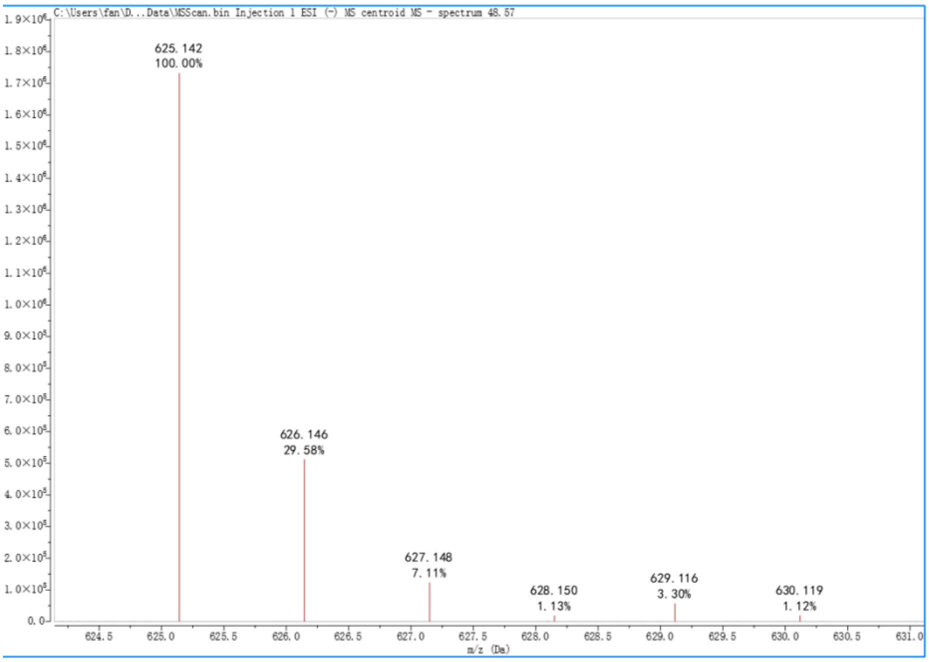


**Figure S52.** The first-order mass spectrum of compound Ⅳ

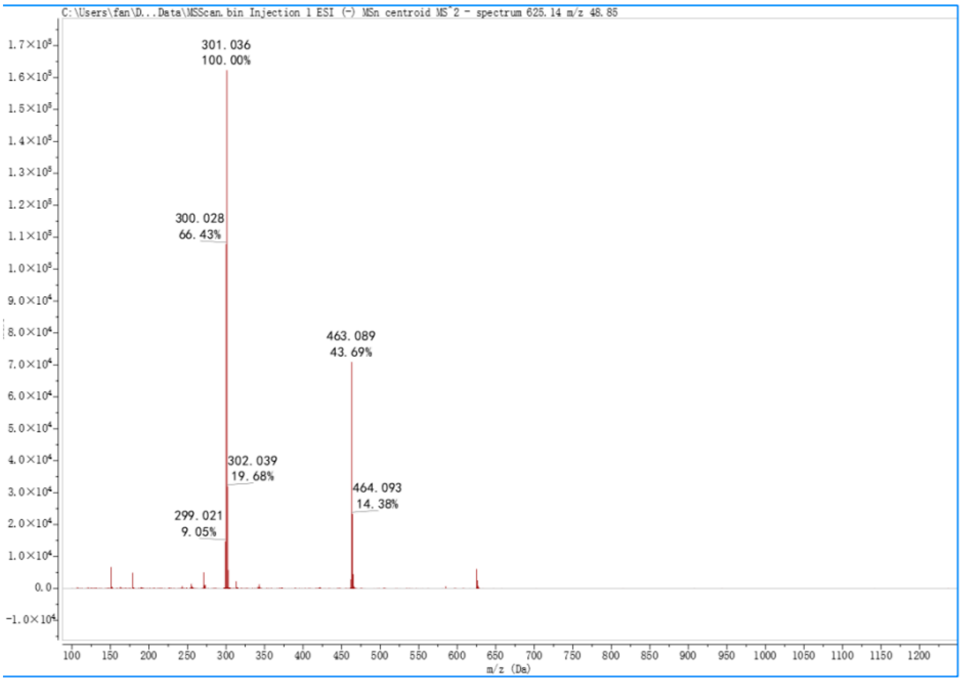


**Figure S53.** Secondary mass spectrum of compound Ⅳ

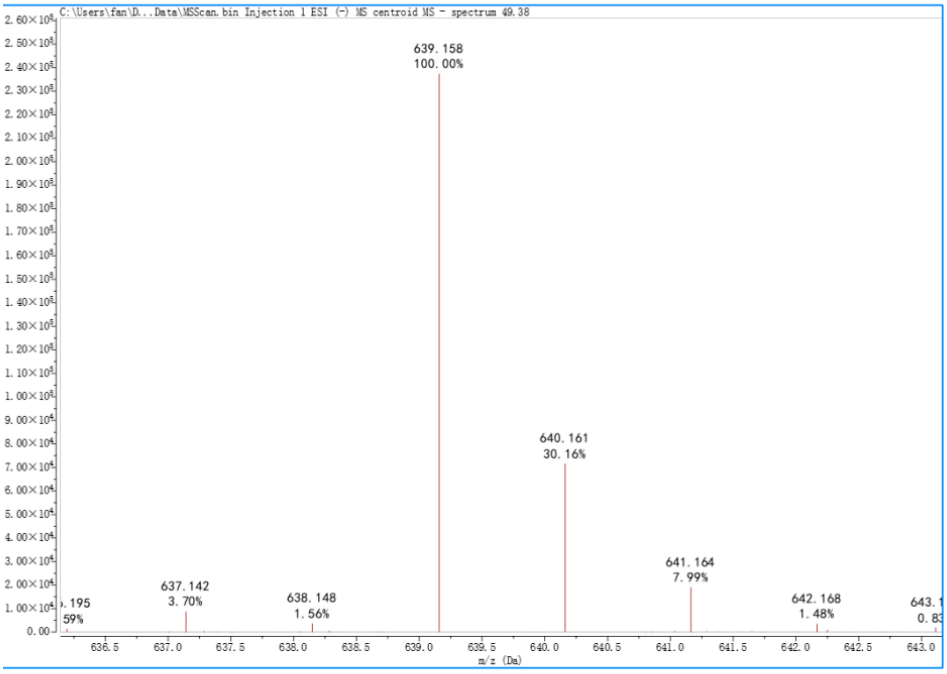


**Figure S54.** The first-order mass spectrum of compound Ⅴ

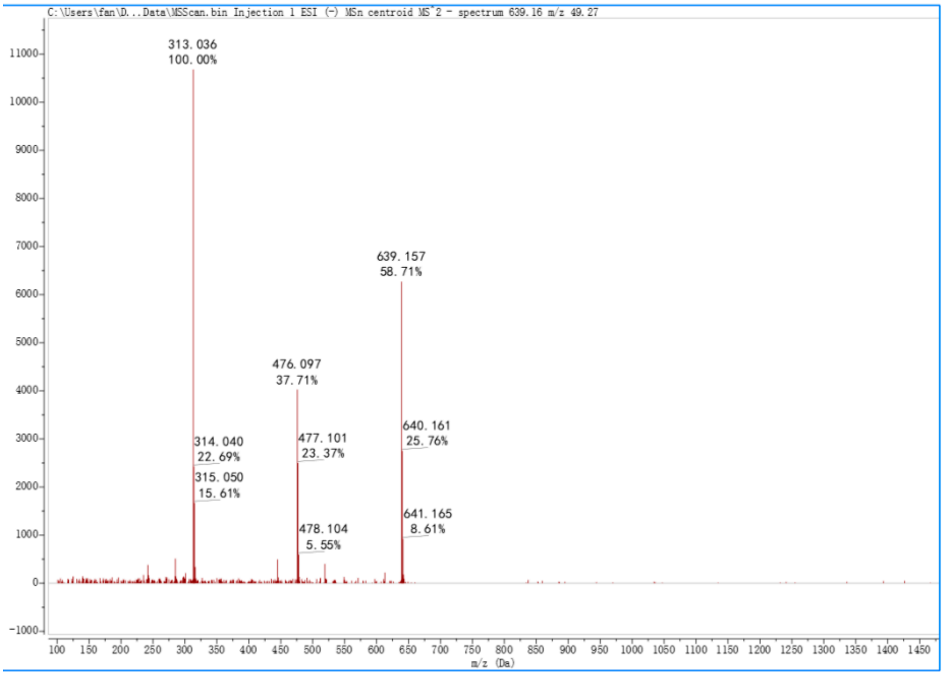


**Figure S55.** Secondary mass spectrum of compound Ⅴ

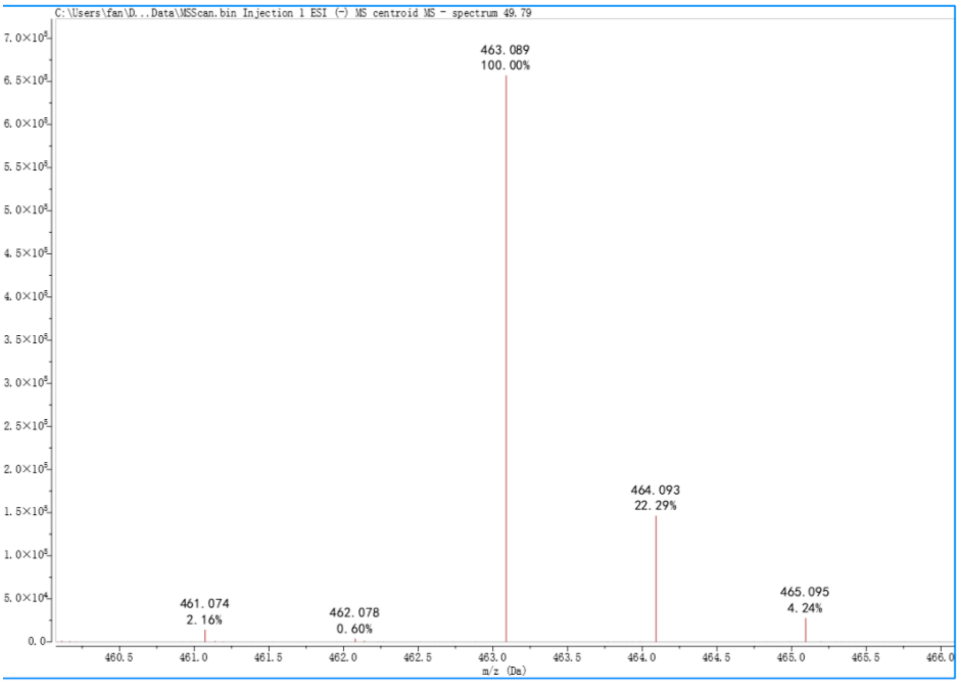


**Figure S56.** The first-order mass spectrum of compound Ⅵ

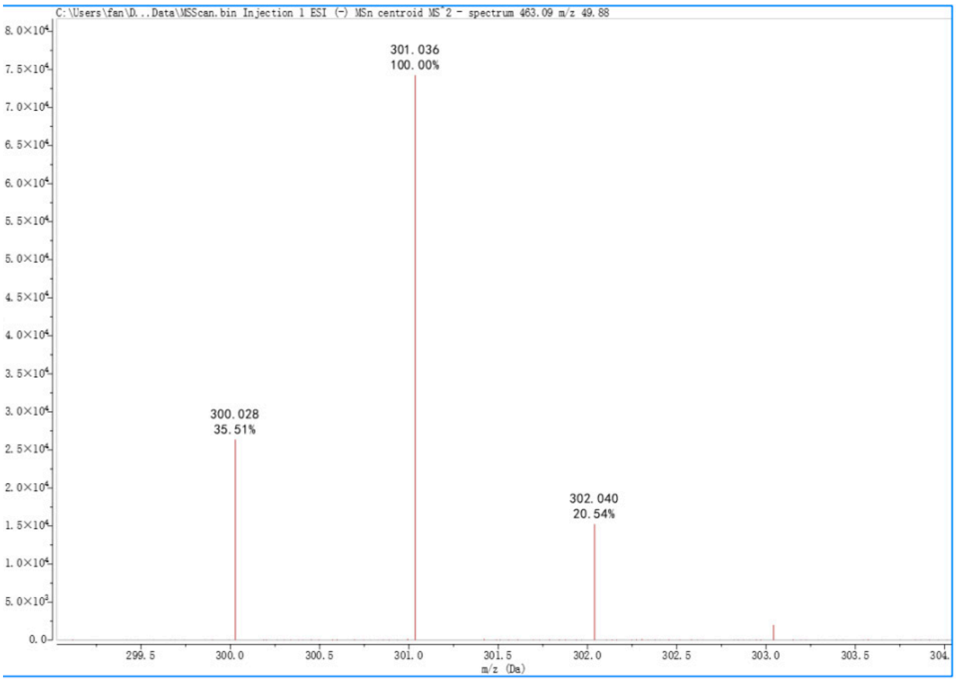


**Figure S57.** Secondary mass spectrum of compound Ⅵ

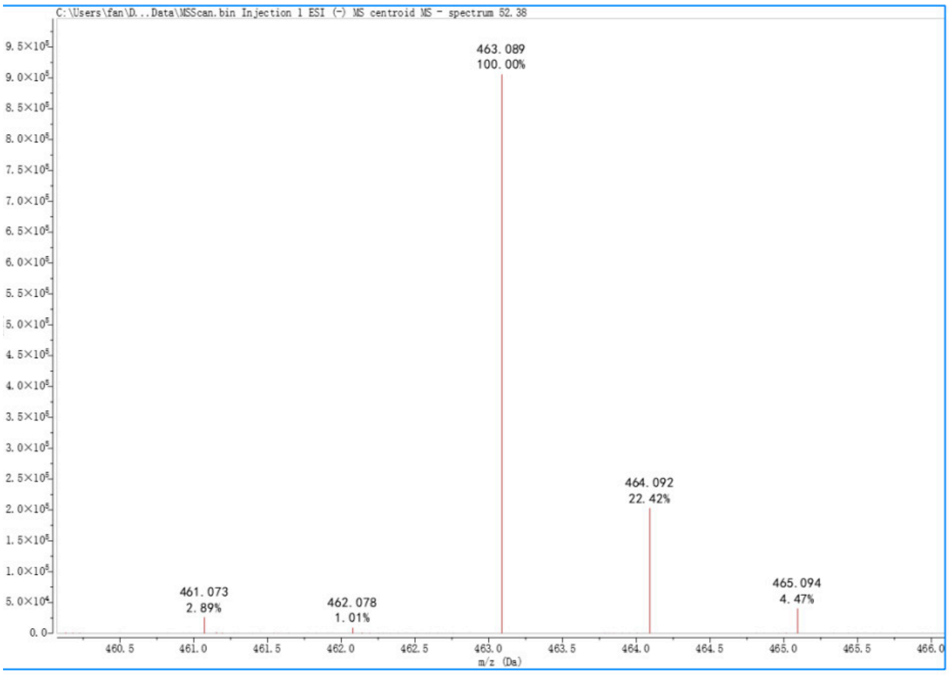


**Figure S58.** The first-order mass spectrum of compound Ⅶ

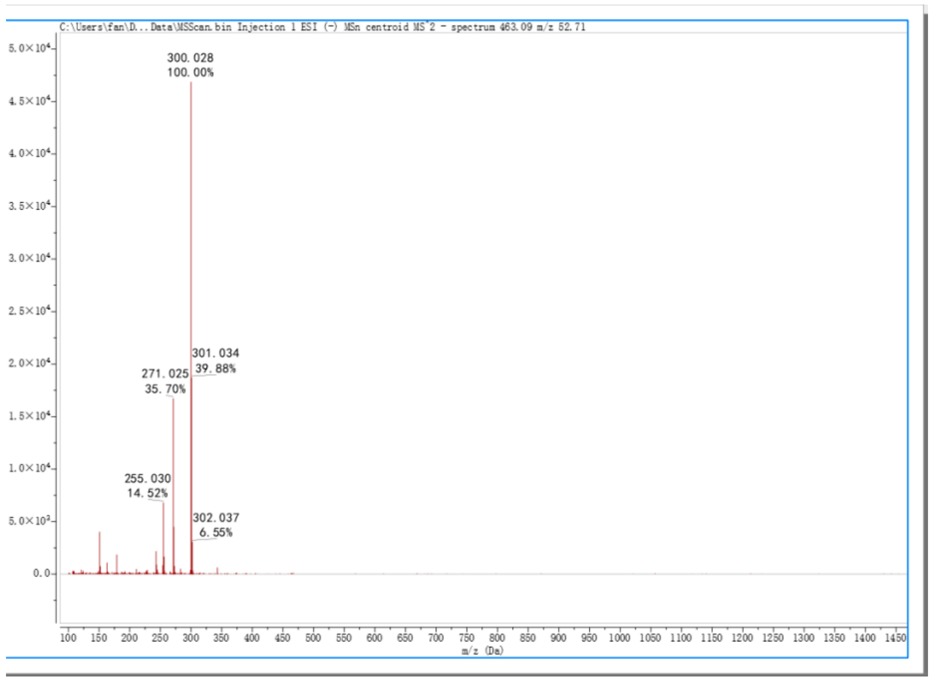


**Figure S59.** Secondary mass spectrum of compound Ⅶ

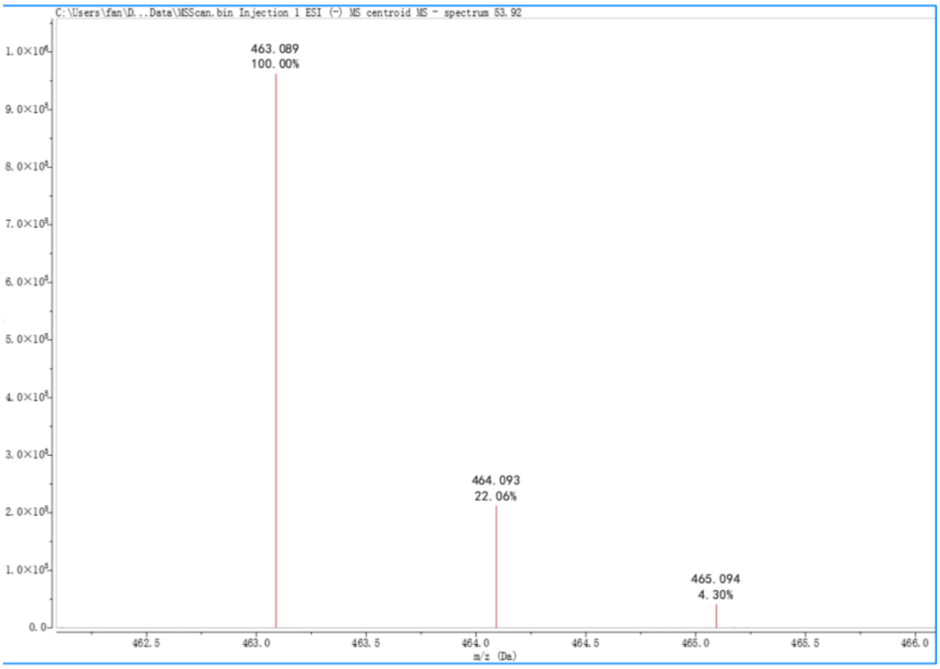


**Figure S60.** The first-order mass spectrum of compound Ⅷ

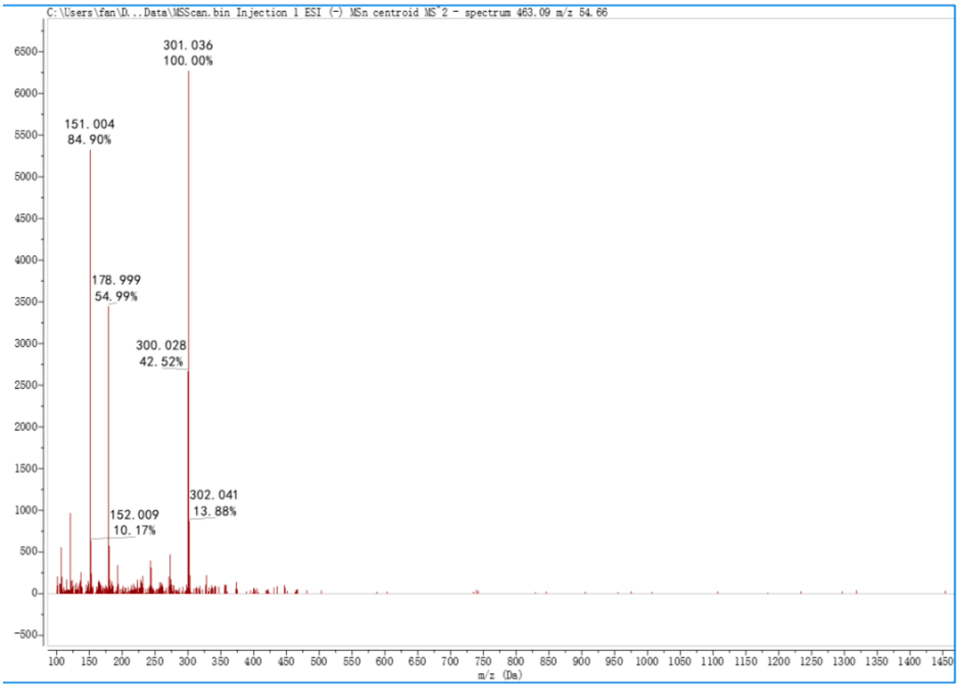


**Figure S61.** Secondary mass spectrum of compound Ⅶ

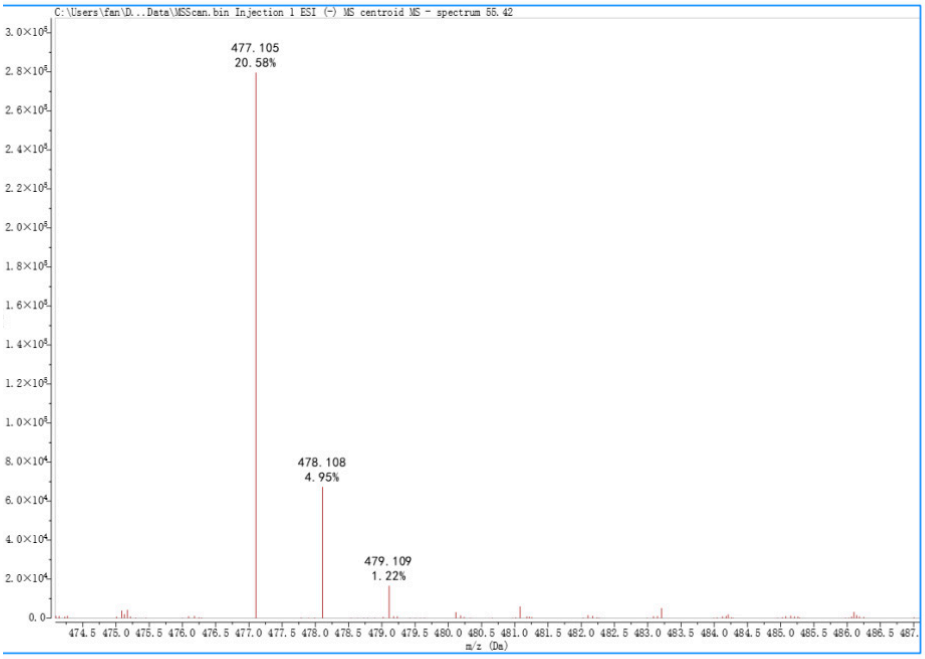


**Figure S62.** The first-order mass spectrum of compoundⅨ

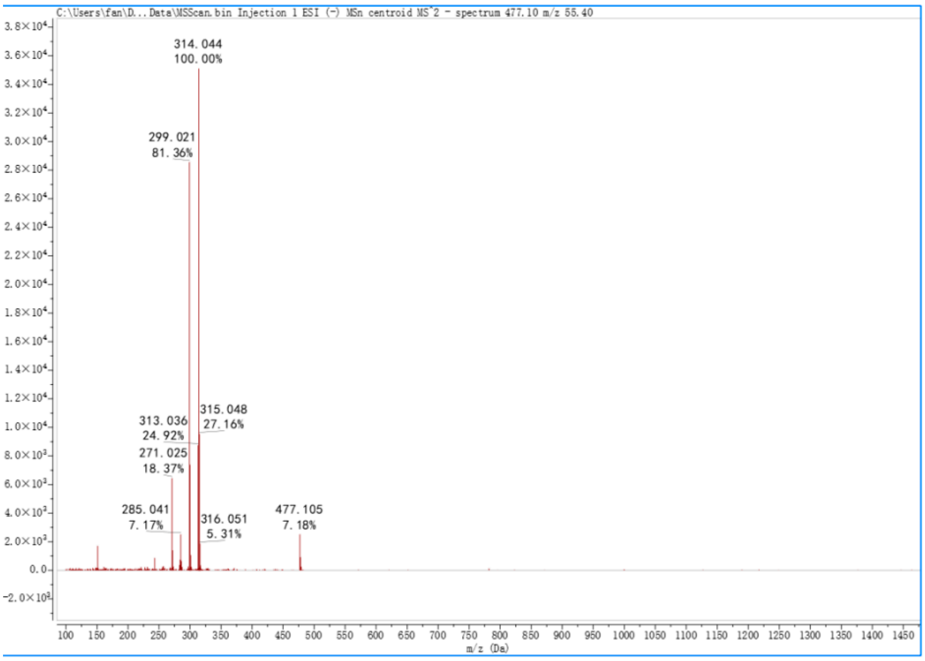


**Figure S63.** Secondary mass spectrum of compound Ⅸ

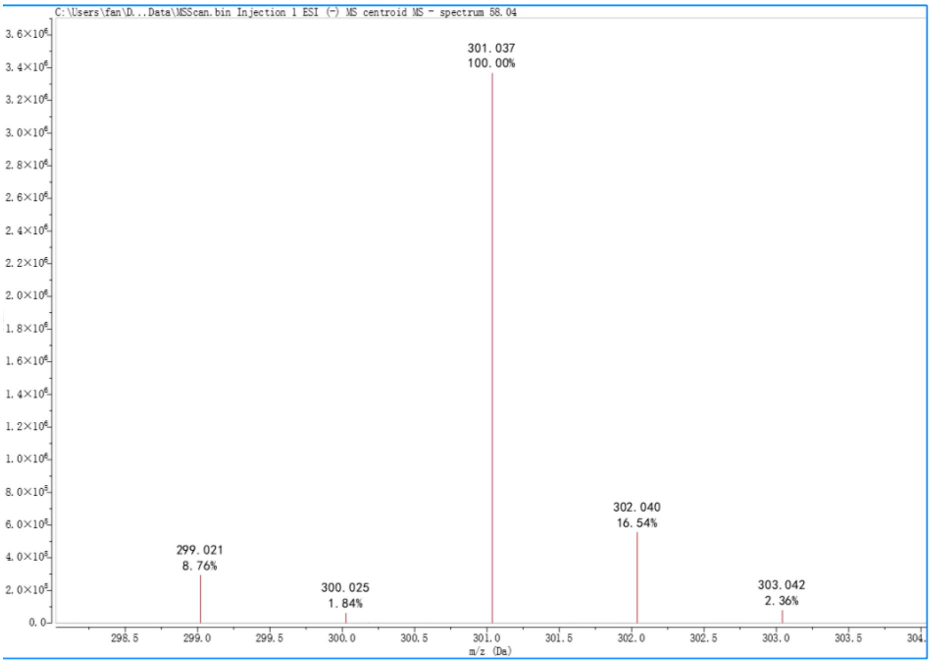


**Figure S64.** The first-order mass spectrum of compound Ⅹ

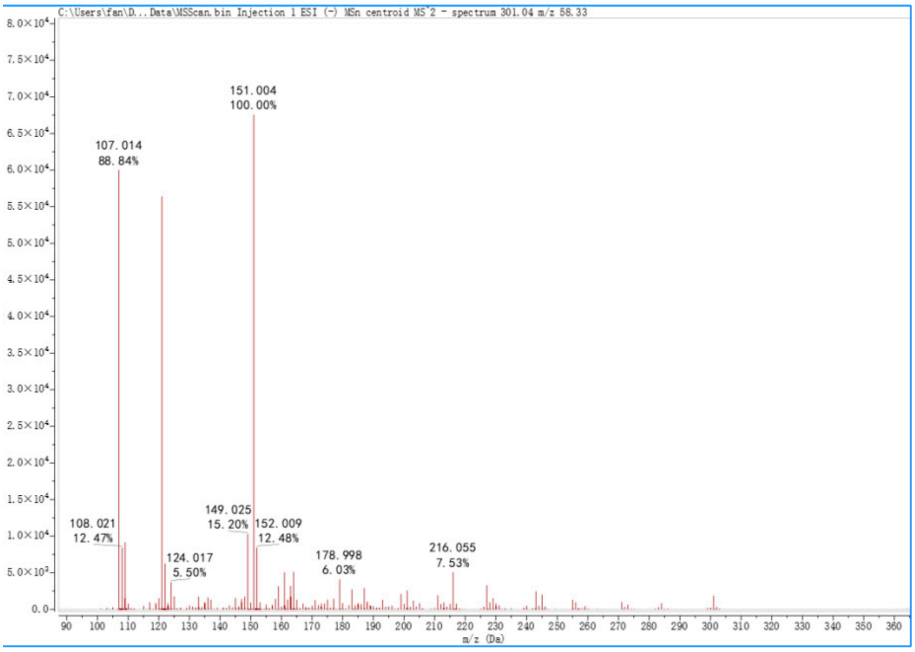


**Figure S65.** Secondary mass spectrum of compound Ⅹ
